# Dynamics of target-mediated drug disposition: characteristic profiles and parameter identification

**DOI:** 10.1007/s10928-012-9260-6

**Published:** 2012-08-01

**Authors:** Lambertus A. Peletier, Johan Gabrielsson

**Affiliations:** 1 Mathematical Institute, Leiden University, PB 9512, 2300 RA Leiden, The Netherlands; 2 Division of Pharmacology and Toxicology, Department of Biomedical Sciences and Veterinary Public Health, Swedish University of Agricultural Sciences, Box 7028, 750 07 Uppsala, Sweden

**Keywords:** Target, Receptor, Antibodies, Drug-disposition, Michaelis–Menten, Quasi-steady-state, Quasi-equilibrium, Singular perturbation

## Abstract

In this paper we present a mathematical analysis of the basic model for target mediated drug disposition (TMDD). Assuming high affinity of ligand to target, we give a qualitative characterisation of ligand versus time graphs for different dosing regimes and derive accurate analytic approximations of different phases in the temporal behaviour of the system. These approximations are used to estimate model parameters, give analytical approximations of such quantities as area under the ligand curve and clearance. We formulate conditions under which a suitably chosen Michaelis–Menten model provides a good approximation of the full TMDD-model over a specified time interval.

## Introduction

The interaction of ligand and target in the process of drug-disposition offers interesting examples of complex dynamics when target is synthesised and degrades and when both ligand and ligand–target complex are eliminated. In recent years such dynamics has received considerable attention because it is important in the context of data analysis, but also, more generally, in the context of system biology because this model serves as a module in more complex systems [1].

Based on conceptual ideas developed by Levy [2], the basic model for target mediated drug disposition (TMDD) was formulated by Mager and Jusko [3]. Earlier studies of ligand–target interactions go back to Michaelis and Menten [4]. We also mention ideas about receptor turnover developed by Sugiyama and Hanano [5]. Mager and Krzyzanski [6] showed how rapid binding of ligand to target leads to a simpler model, Gibiansky et al. [7] studied the related quasi-steady-state approximation to the model and Marathe et al. [8] conducted a numerical validation of the *rapid binding* approximation. Gibiansky et al. [9] also pointed out a relation with the classical indirect response model. For further background we refer to the books by Meibohm [10] and Crommelin et al. [11], and to the reviews by Lobo et al. [12] and Mager [13].

In practice, the Michaelis–Menten model is often used when ligand curves exhibit TMDD characteristics (see e.g. Bauer et al. [14]). Recently, Yan et al. [15] analysed the relationship between TMDD- and Michaelis–Menten type dynamics. We also mention the work by Krippendorff et al. [16] which studies an extended TMDD system which includes *receptor trafficking* in the cell.

The characteristic features of TMDD dynamics were first studied in [3] under the condition of a constant target pool, i.e., the total amount of target: free and bound, was assumed to be constant in time. Under the same assumption, a mathematical analysis of this model was offered by Peletier and Gabrielsson [17]. This assumption was made, in part for educational reasons, because it makes a transparent geometric description possible, in which qualitative and quantitative properties of the dynamics can be identified and illustrated. In a recent paper Ma [18] compared different approximate models under the same assumption of a constant target pool.

In the present paper we extend this analysis to the *full* TMDD model and do not make the assumption that the target pool is constant. This means, in particular, that we shall now also be enquiring as to how the target pool changes over time and how it is affected by the dynamics of its zeroth order synthesis and first order degeneration.

The analysis in this paper consists of a combination of numerical simulations based on a specific case study, and a detailed mathematical analysis of the set of three differential equations that constitute the full TMDD model. Our main objective will be to gain insight in such issues as:

1. *Properties of concentration profiles* When the initial ligand concentration is larger than the endogenous receptor concentration, the dynamics of target-mediated drug disposition results in a characteristic ligand versus time profile. In Fig. 1 we show such a profile schematically.

**Fig. 1:**
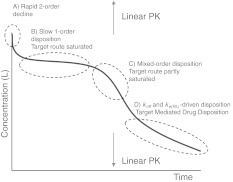
Characteristic ligand versus time graph in target-mediated drug disposition. The concentration of the ligand is measured on a logarithmic scale. In the first phase (*A*) drug and target rapidly equilibrate, in the second phase (*B*) the target is saturated and drug is mainly eliminated directly by a first order process, in the third phase (*C*) the target is no longer saturated and drug is eliminated directly, as well as in the form of a drug–target complex, and in the final, fourth phase (*D*) the drug concentration is so low that elimination is a linear first order process with direct as well as indirect elimination (as a drug–target complex)
  One can distinguish four different phases in the dynamics of the system in which different processes are dominant: (A) a brief initial phase, (B) an apparent linear phase, (C) a transition phase and (D) a linear terminal phase. We obtain precise estimates for the duration of each of these phases and for each of them we obtain accurate analytical estimates for the concentration versus time graphs of the ligand, the receptor and the ligand–receptor complex.
  On the basis of ligand concentration versus time curves we will develop instruments for extracting information about the target and the ligand–target complex versus time curves.
2. *Parameter identifiability* We shed light on what we can predict when we have only measured (a) the ligand, (b) ligand and target, (c) ligand and complex, and (d) all of the above.
3. *Systems analysis* Whilst focussing on concentration versus time curves we gain considerable qualitative understanding and quantitative estimates about the impact of the different parameters in the model and on quantities such as the area under the curve and time to steady state of the different compounds.
4. *Model comparison* An important issue is the question as to how the full TMDD model compares with the simpler Michaelis–Menten model [7, 8, 15]. In this paper we point out how the full model and the reduced Michaelis–Menten model differ significantly in the initial second order phase and in the linear terminal phase, in that the terminal rate (λ_*z*_) of ligand in the full model is much smaller than that in the Michaelis–Menten model.

**Fig. 2:**
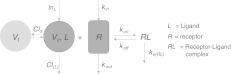
Schematic description of target-mediated drug (or ligand) disposition. The ligand *L* binds reversibly (*k*_on_/*k*_off_) to the target *R* to form the ligand–target complex *RL*, which is irreversibly removed via a first order rate process (*k*_*e*(*RL*)_), and in addition is eliminated via a first order process (*k*_*e*(*L*)_ = *Cl*_(*L*)_/*V*_*c*_)

In this paper we focus on the classical TMDD model, as presented in for instance [3] and shown schematically in Fig. 2. However, since we will focus on the typical features of the interaction between the ligand *L*, the receptor *R* and their complex *RL*, we drop the peripheral compartment (*V*_*t*_, *Cl*_*d*_) of the ligand. In mathematical terms, the model then results in the following system of ordinary differential equations:

1 $$ \left\{ \begin{array}{ll} \frac{dL}{dt} &= k_f -k_{\rm on} L \cdot R + k_{\rm off} RL - k_{e(L)}L \\ \frac{dR}{dt} &= k_{\rm in} - k_{\rm out} R -k_{\rm on} L \cdot R + k_{\rm off} RL \\  \frac{dRL}{dt}&= k_{\rm on} L \cdot R - (k_{\rm off}+ k_{e(RL)})RL \end{array}\right. $$

The quantities *L*, *R* and *RL* are assumed to be concentrations, *k*_*f*_ = *In*_*L*_/*V*_*c*_ denotes the infusion rate of the ligand (here *In*_*L*_ denotes the infusion of ligand and *V*_*c*_ the volume of the central compartment), and *k*_on_ and *k*_off_ denote the second-order on- and first-order off rate of the ligand. Ligand is eliminated according to a first order process involving the rate constant *k*_*e*(*L*)_ = *Cl*_(*L*)_/*V*_*c*_, where *Cl*_(*L*)_ denotes the clearance of ligand from the central compartment. Ligand–target complex leaves the system according to a first order process with a rate constant *k*_*e*(*RL*)_. Finally, receptor synthesis and degeneration are, respectively, a zeroth order (*k*_in_) process and a first order (*k*_out_) process.

In the absence of a zeroth-order infusion of ligand, i.e., when *k*_*f*_ = 0, the steady state of the system (1) is given by

2 $$ L=0, \quad R=R_0 \mathop{=}\limits^{{\rm def}} \frac{k_{\rm in}}{k_{\rm out}}, \quad RL=0 $$

Thus, this is the situation when there is no free or bound ligand. The receptor concentration then satisfies a simple turnover equation involving zeroth order synthesis and first order degeneration:

$$ \frac{dR}{dt} =k_{\rm in} - k_{\rm out} \cdot R $$

with steady state *R*_0_ = *k*_in_/*k*_out_.

The TMDD system can be viewed as one in which two constituents, the ligand and the target, or receptor, are interacting with one another whilst each of them is supplied and removed, either in their free form, or in combination in the form of a complex. We shall find that the total quantities of ligand and receptor will play a central role. Therefore, we put

3 $$ L_{\rm tot} = L + RL \quad \hbox {and} \quad R_{\rm tot} = R + RL $$

We deduce from the system (1) that their behaviour with time is given by the following pair of *conservation laws*:

4 $$ \left\{ \begin{array}{l} \frac{dL_{\rm tot}}{dt} = k_f - k_{e(L)}L - k_{e(RL)} RL \quad \hbox {for\,the\,ligand}\,L \\ \frac{dR_{\rm tot}}{dt} = k_{\rm in} - k_{\rm out} R - k_{e(RL)} RL \quad \hbox {for\,the\,receptor}\,R \end{array}\right. $$

Note that in this system the on- and off rates of ligand to receptor no longer appear.

We investigate the dynamics of the system (1) that is generated by two types of administration of the ligand:

- (i) Through a bolus dose. Then *k*_*f*_ = 0. We denote the initial ligand concentration by *L*(0) = *L*_0_ = *D*/*V*_*c*_, where *D* is the dose and *V*_*c*_ the volume of the central compartment.
- (ii) Through a constant rate infusion *In*_*L*_. Then *k*_*f*_ = *In*_*L*_/*V*_*c*_ > 0 and *L*(0) = 0.

When we assume that prior to administration the system is at baseline, the initial values of ligand, receptor and ligand–receptor complex will be

5 $$ L(0)=L_0 \hbox{(bolus) or}\,L(0)=0\,\hbox {(infusion)}, \quad R(0)=R_0, \,\, RL(0)=0 $$

In this paper we focus on the situation when *k*_*e*(*RL*)_, *k*_off_ and *k*_*e*(*RL*)_ are small compared to *k*_on_ *R*_0_.

Whereas in our earlier investigation [17], the total amount of receptor was constant, because it was assumed that *k*_*e*(*RL*)_ = *k*_out_, here this assumption is no longer made and, generally, *R*_tot_ will vary with time. However, we show that there exists an upper bound for the total amount of receptor in the system, free and bound, which is independent of the amount of ligand supplied and holds for both types of administration. Specifically, we prove that, starting from baseline,

6 $$ R_{\rm tot}(t) \le {\rm max}\{R_0,R_*\} \quad \hbox {for all} \, t \ge 0 \quad \hbox {where} \, R_*=\frac{k_{\rm in}}{k_{e(RL)}} $$

For the proof of this bound we refer to Appendix 3.

Anchoring our investigation in a case study in which ligand is administered through a series of bolus doses, we dissect the resulting time courses of the three compounds, *L*, *R* and *RL* and identify characteristic phases, *Phases A–D* shown in Fig. 1. We associate these phases with specific processes and show, using *singular perturbation theory* [19, 20, 21], that individual phases may be analysed through appropriately chosen simplified models, yielding accurate closed-form approximations. They offer tools which may be used to compute critical quantities such as *residence time*, and to verify whether different approximations to the full TMDD model, such as the *rapid binding approximation* [6] and the *quasi-steady-state approximation* [7, 18] are valid in the different phases. These issues are discussed at the conclusion of this paper.

Much of the mathematical analysis underpinning the results presented throughout the text is presented in a series of Appendices at the end of the paper.

## Case study

The ligand, target and complex concentration–time courses used throughout this analysis, were simulated to mimic real experimental observations obtained on a monoclonal antibody dosed to marmoset monkeys. Simulated data were generated with a constant coefficient of variation (2 %) of the error added to the data. Synthetic data were used for pedagogic and proprietary reasons in order to answer the question: ”To what extent is the parameter precision affected by including/not including target (*R*) and complex (*RL*) data. The model used for generating the data is shown in Fig. 2 and the actual parameters are stored in Table 1. WinNonlin 5.2, with a Runge–Kutta–Fehlberg differential equation solver, was used for both simulating and regressing data. A constant CV (proportional error) model was used as weighting function. All dose levels (Concentration–time courses) were simultaneously regressed for the ligand (*L*), ligand and target (*L*, *R*) and ligand, target and complex (*L*, *R* and *RL*) data analyses, respectively. Kinetic data of high quality—as regards spacing in time and concentration and very low error level—were used intentionally to demonstrate (a) improved precision when using two or more sources of chemical entities; (b) as a check that one gets back approximately the same parameter estimates as used for generating the original dataset(s).

**Table 1:** Pre-selected parameter values

Symbol	Unit	Value
*V*_*t*_	L/kg	0.1
*Cl*_*d*_	(L/kg)/h	0.003
*Cl*_(*L*)_	(L/kg)/h	0.001
*k*_on_	(mg/L)^−1^/h	0.091
*k*_off_	1/h	0.001
*k*_in_	(mg/L)/h	0.11
*k*_out_	1/h	0.0089
*k*_*e*(*RL*)_	1/h	0.003
*R*_0_	mg/L	12

### Data analysis

**Fig. 3:**
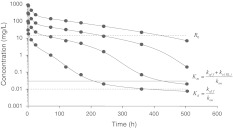
Semi-logarithmic graphs of the ligand plasma concentration versus time after the administration of four rapid intravenous injections *D* of 1.5, 5, 15 and 45 mg/kg, respectively (Data set (I)). The volume of the central compartment *V*_*c*_ for these doses was fixed at 0.05 L/kg. The *dots* are simulated data and the *solid curves* are obtained by fitting the model sketched in Fig. 2 to the data. Estimates are given in Table 2

**Fig. 4:**
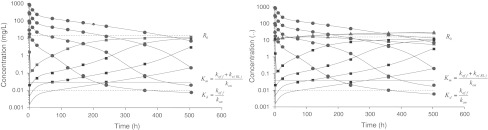
*Left* semi-logarithmic graphs of simulated plasma concentrations of *L* (*red discs*) and *R* (*blue squares*) versus time (Data set (II)) and on the *right* the same, but also semi-logarithmic graphs of *RL* (*green triangles*) (Data set (III)), taken after administration of four rapid intravenous injections *D* of 1.5, 5, 15 and 45 mg/kg, respectively. *V*_*c*_ for these doses was fixed at 0.05 L/kg. The *dots* are simulated data and the *solid curves* are obtained by fitting the model sketched in Fig. 2 to the data. Estimates are given in Table 2

The case study is based on three sets of simulated concentration versus time data (I, II and III), each set of data obtained after following four rapid intravenous injections of the ligand or antibody (*L*). These datasets are shown in Figs. 3 and 4. They are increasing in richness: the first set (I) contains ligand profiles only, the second set (II) contains ligand as well as target or receptor profiles and the third set (III) contains profiles of all three compounds: the ligand, the receptor and the ligand–receptor complex.

The purpose of this study is to demonstrate the possibility of fitting the eight-parameter model shown in Fig. 2, to three different sets of high quality data with increasing richness, and show how precision of the estimates of the model parameters increases when successively information about target (II) and target and complex (III) is added. We use this data set for two purposes: (i) for data analysis and (ii) for highlighting critical features of the temporal behaviour of the three compounds.

Simulated data from three sources (ligand, target and complex) were intentionally used. We have experienced that data of less quality gave biased and imprecise estimates as well as biased and imprecise predictions of ligand, target and complex.

The central volume *V*_*c*_ was assumed to be equal to 0.05 L/kg and fixed. The other parameters are then re-estimated for datasets I–III (Table 2). Thus, we know a priori what values they should have.

**Table 2:** Final parameter estimates and their relative standard deviation (CV%) on the basis of the three datasets

Symbol	Unit	I (*L*)	II (*L* & *R*)	III (*L* & *R* & *RL*)
*V*_*t*_	L/kg	0.101 (2)	0.100 (2)	0.100 (1)
*Cl*_*d*_	(L/kg)/h	0.003 (4)	0.003 (3)	0.003 (3)
*Cl*_(*L*)_	(L/kg)/h	0.001 (1)	0.001 (1)	0.001 (1)
*k*_on_	(mg/L)^−1^/h	0.099 (17)	0.092 (2)	0.096 (1)
*k*_off_	1/h	0.001 (27)	0.001 (13)	0.001 (3)
*k*_out_	1/h	0.009 (6)	0.009 (2)	0.009 (2)
*k*_*e*(*RL*)_	1/h	0.002 (27)	0.002 (23)	0.002 (2)
*R*_0_	mg/L	12 (4)	12 (1)	12 (1)

Dataset I is made up from simulated concentration–time profiles covering five orders of magnitude in concentration range and from 0 to 500 h. Dataset II contains the same simulated ligand (*L*) profiles as in dataset I as well as target (*R*) concentration–time profiles obtained at each dose level. Dataset III includes dataset II but is enriched by four simulated time-courses of the ligand–target complex (*LR*) as well.

The four doses are *D* = 1.5, 5, 15 and 45 mg/kg. The volume *V*_*c*_ of the central compartment being 0.05 L/kg, this yields the following initial ligand concentrations *L*_0_ = *D*/*V*_*c*_ = 30, 100, 300 and 900 mg/L.

The parameter values given in Table 1 yield the following values for the dissociation constant *K*_*d*_ and the constant *K*_*m*_ related to *K*_*d*_:

7 $$ K_d = \frac{k_{\rm off}}{k_{\rm on}} = 0.011 \,\hbox {mg/L} $$

and

8 $$ K_m = \frac{k_{\rm off} + k_{e(RL)}}{k_{\rm on}} = 0.044 \,\hbox{mg/L} $$

Here, *K*_*d*_ is a measure of affinity between drug (ligand) and target, whereas *K*_*m*_ is more of a conglomerate of affinity (*K*_*d*_) and irreversible elimination of the ligand–target complex (*k*_*e*(*RL*)_) and used for comparisons to the Michaelis–Menten parameter *K*_*M*_ of regression model Eq. (45). Unless the removal of the ligand–target complex is fully understood, one should be careful about the interpretation of an apparent *K*_*m*_-value. *K*_*m*_ can be very different from the affinity *K*_*d*_. Here, solid biomarker (physiological or disease markers) data on effective plasma concentrations may be a practical guidance.

Summarizing we may conclude that:

- Dataset I—which involves *L*—allows the prediction of robust ligand concentration–time profiles within the suggested concentration and time frame. We see that if only ligand data are available, the majority of parameters except for *k*_on_, *k*_off_ and *k*_*e*(*RL*)_ are estimated with high precision. The latter three parameters are still highly dependent on information about the time courses of either target and/or complex. Since *k*_kon_, *k*_off_ and *k*_*e*(*RL*)_ have low precision (high CV%) we would discourage the use of these parameters for the prediction of tentative target and complex concentrations.
- Dataset II—which involves *L* and *R*—still gives good precision in all parameters except *k*_off_ and *k*_*e*(*RL*)_, which will also be highly correlated. Since we also have experimental data of the target we encourage the use of this model for interpolation of target concentration–time courses, but not for concentration–time courses of the complex.
- Dataset III—which includes *L* and *R*, as well as *RL*—gives high precision in all parameters. Since we also have measured the complex concentration–time course with high precision we obtained *k*_off_ and *k*_*e*(*RL*)_ values with high precision. We doubt that the practical experimental situation can get very much better than this latter case where we have simultaneous concentration–time courses of *L*, *R* and *RL* with little experimental error due to biology and bio-analytical methods. Dataset III is an ideal case; the true experimental situation seldom gets better.

We also doubt the practical value of regressing too elaborate models to data. Models that capture the overall trend nicely but result in parameters with low precision and biased estimates may be of little value.

The volume of the central compartment *V*_*c*_ ought to fall somewhere in the neighbourhood of the plasma water volume (0.05 L/kg) for large molecules in general and antibodies in particular. In our own experience of antibody projects this has been the case when data contained an acceptable granularity within the first couple of hours after the injection of the test compound. Therefore we assumed *V*_*c*_ to be a constant term (0.05 L/kg) in this analysis and not part of the list of parameters to be estimated. We think this increases the robustness of the estimation procedure and is biologically viable.

### Critical features of the graphs

The graphs in Figs. 3 and 4 exhibit certain characteristic features and so reveal typical properties of the dynamics of the TMDD system.

- Initially all the ligand graphs in Fig. 3 exhibit a rapid drop which increases in relative sense as the ligand dose decreases. Over this initial period, which we refer to as *Phase A*, (cf. Fig. 1), *R*(*t*) exhibits a steep drop that becomes deeper as the drug dose increases.
- After the brief initial adjustment period, the graphs for large doses reveal linear first order kinetics over a period of time (*Phase B*) that shrinks as the drug dose decreases. At the lowest dose the linear period has vanished and the graph exhibits nonlinear kinetics.
- For the larger doses, there is an upward shift of the linear phase that appears to be linearly related to the ligand dose; the slope of this linear phase appears to be dose-independent.
- The point of inflection in the log(*L*) versus time curve—the middle of *Phase C*—which we observe in the graphs for *L*_0_ = 100 and 300, moves to the right as the initial dose increases, but stays at the same level. This is clearly seen in Fig. 3 in which the baseline value *R*_0_ and the value of *K*_*d*_ and *K*_*m*_ are also shown.
- For the lower doses we see that the log(*L*) versus time curve eventually becomes linear again, with a slope that is markedly smaller than it was in the nonlinear *Phase C* that preceded it. This part of the graph corresponds to *Phase D* in Fig. 1.

Summarising, in the ligand graphs of Fig. 3 we see for the higher drug doses the different phases *A*–*D* that were pointed out in Fig. 1. In the following analysis we explain these features and quantify them in that, for instance, we present estimates for the upward shift referred to in (c) and the right-ward shift of the inflection point alluded to in (d).

## Dynamics after a bolus administration

Here we assume that ligand is supplied through an intravenous bolus administration and that there is no infusion, i.e., *k*_*f*_ = 0. Thus, we focus on the system

9 $$ \left\{ \begin{array}{l} \frac{dL}{dt} = -k_{\rm on} L \cdot R + k_{\rm off} RL - k_{e(L)}L \\ \frac{dR}{dt} = k_{\rm in} - k_{\rm out} R -k_{\rm on} L \cdot R + k_{\rm off} RL \\ \frac{dRL}{dt} = k_{\rm on} L \cdot R - (k_{\rm off}+ k_{e(RL)})RL \\ \end{array}\right. $$

In order to obtain a first impression of typical concentration versus time courses for the three compounds, we carry out a few simulations of the system (9). We then present a mathematical analysis in which we delineate and discuss the four phases *A*, *B*, *C* and *D* in the characteristic ligand versus time graph shown in Fig. 1.

### Simulations

We use the same initial doses as in the case study, i.e., the initial ligand concentrations are *L*_0_ = 30, 100, 300 and 900 mg/L. The parameter values are given in Table 3, which is the same as Table 1, except that (i) the parameters *V*_*T*_ and *Cl*_*d*_ are absent because the tissue compartment has been taken out and (ii) the elimination rate has been reduced to *k*_*e*(*L*)_ = 0.0015 h^−1^ so that the different phases in the ligand versus time graphs can be distinguished more clearly.

**Table 3:** Parameter estimates used for demonstrating the dynamics of *L*, *R* and *RL* after bolus and constant-rate infusion regimens of *L*

Symbol	Unit	Value
*k*_*e*(*L*)_	1/h	0.0015
*k*_on_	(mg/L)^−1^/h	0.091
*k*_off_	1/h	0.001
*k*_in_	(mg/L)/h	0.11
*k*_out_	1/h	0.0089
*k*_*e*(*RL*)_	1/h	0.003
*R*_0_	mg/L	12

**Fig. 5:**
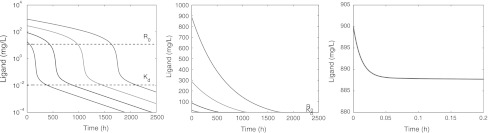
Graphs of *L* versus time on semi-logarithmic scale (*left*), on a linear scale (*middle*) and a close up (*right*), for doses resulting in initial ligand concentrations *L*_0_ = 30, 100, 300, 900 mg/L and parameters listed in Table 3. In addition, *R*(0) = *R*_0_ and *RL*(0) = 0. The *dashed lines* indicate the target baseline level *R*_0_, and the dissociation constant *K*_*d*_

In Fig. 5 we show three ligand versus time graphs. In the figure on the left, *L* is given on a logarithmic scale and in the two figures on the right *L* is given on a linear scale, with the one on the right a blow-up of the initial behaviour when *L*_0_ = 900 mg/L.

The ligand concentration curves shown in the left panel of Fig. 5 exhibit the characteristic shape shown in Fig. 1. They consist of the following segments:

- A rapid initial adjustment (see the blow-up on the right).
- A first linear phase with a slope which is independent of the dose, and which shifts upwards as the drug dose increases.
- A transition phase which shifts to the right as the drug dose increases, but maintains its level.
- A final linear terminal phase with a slope λ_*z*_ that is again independent of the drug dose. For the parameter values of Table 3 we find that λ_*z*_ ≈ *k*_*e*(*RL*)_ = 0.003.

Since in Figs. 3 and 4 the time was restricted to 500 h, the terminal phase (*D*) has only just begun for the lowest dose, and not even started for the higher doses.

**Fig. 6:**
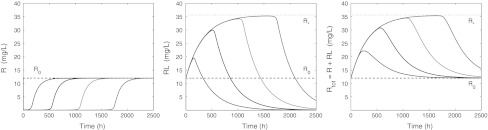
Graphs of *R* (*left*), *RL* (*middle*) and *R*_tot_ (*right*) versus time for *L*_0_ = 30, 100, 300, 900 mg/L and parameters given in Table 3, whilst *R*(0) = *R*_0_ and *RL*(0) = 0. The *dashed line* indicates the target baseline level *R*_0_ and the *dotted line* the level R_*_

In Fig. 6 we present the **receptor dynamics**: concentration versus time profiles for, respectively, *R*, *RL* and *R*_tot_ when *L*_0_ > *R*_0_. Whereas in [17] it was assumed that *k*_out_ = *k*_*e*(*RL*)_, and hence the receptor pool was constant (*R*_tot_ ≡ *R*_0_), for the parameters in Table 3 we have *k*_out_ > *k*_*e*(*RL*)_, so that *R*_tot_ is no longer constant.

We make the following observations:

- (i) The total amount of target *R*_tot_(*t*)—free and bound to ligand—increases from its initial value *R*_0_ = 12 to a maximum value R_*_, and then drops off again towards its baseline value *R*_0_. A similar observation was made in [15, 22].

We shall prove that

10 $$ R_* = \frac{k_{\rm in}}{k_{e(RL)}} $$

Thus, if *k*_*e*(*RL*)_ < *k*_out_, as is the case with the parameter values of Table 3, then R_*_ > *R*_0_ and the total target pool *increases* before it returns to the baseline value *R*_0_.

Alternatively, if *k*_*e*(*RL*)_ > *k*_out_, then R_*_ < *R*_0_ and we show that the total target pool first *decreases* before it returns to the baseline value *R*_0_.

Finally, if *k*_*e*(*RL*)_ = *k*_out_, then *R*_tot_(*t*) = *R*_0_ for all *t* ≥ 0 [3, 17].

- (ii) As the drug dose increases, *R*(*t*) ≈ 0 for an increasing time interval and the graphs of *RL*(*t*) and *R*_tot_(*t*) trace—for the same increasing time interval—a common curve $\Upgamma$ in the (*t*, *R*_tot_)-plane (cf. Fig. 9). This curve $\Upgamma$ is monotonically increasing and tends to the limit R_*_ as $t \to \infty.$ If *k*_*e*(*RL*)_ > *k*_out_, we show that an analogous phenomenon occurs along a curve $\Upgamma,$ which still tends to R_*_, but is now decreasing.

**Fig. 7:**
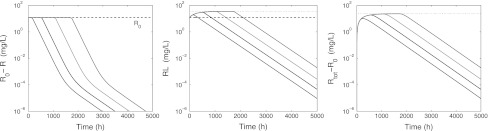
Graphs of *R*_0_ − *R* (*left*), *RL* (*middle*) and *R*_tot_ − *R*_0_ (*right*) versus time on a semi-logarithmic scale for *L*(0) = 30, 100, 300, 900 mg/L and *R*(0) = *R*_0_ and *RL*(0) = 0 mg/L. The parameters are listed in Table 3. In the *middle figure*, the *dashed line* indicates the baseline *R*_0_ and the *dotted line* the level R_*_. In the *right figure* the *dotted line* indicates R_*_ − *R*_0_

In Fig. 7 we present graphs of *R* − *R*_0_, *RL* and *R*_tot_ − *R*_0_ on a logarithmic scale. We note two conspicuous features:

- (i) The three graphs exhibit a *kink* (a sharp angle) which shifts to the right (increasing time) as the drug dose increases.
- (ii) *R*(*t*) − *R*_0_ tends to zero as $t \to \infty$ in a bi-exponential manner, whilst *RL*(*t*) and *R*_tot_(*t*) − *R*_0_ converge to zero in a mono-exponential way.

### Low dose graphs

We conclude these simulations with a comparison of high-dose and low-dose graphs. We do this by adding simulations for initial ligand concentrations which are smaller than *R*_0_. Specifically, we add the values *L*_0_ = 0.3, 1, 3 and 10 mg/L to the graphs shown in Figs. 5 and 6.

**Fig. 8:**
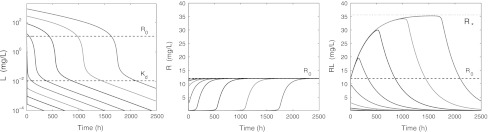
Graphs of *L* on a semi-logarithmic scale (*left*) and *R* (*middle*) and *RL* (*right*) on a linear scale versus time for *L*(0) = 0.3, 1, 3, 10, 30, 100, 300, 900 mg/L and *R*(0) = *R*_0_ and *RL*(0) = 0. The parameters are listed in Table 3. The *dashed lines* indicate the baseline *R*_0_ and *K*_*d*_, and the *dotted line* the level R_*_

Figure 8 shows simulations of the ligand, target and complex concentration–time courses after *eight* different intravenous bolus doses. The initial drop will be difficult to capture unless that is taken care of experimentally within the very first minutes or so when the second-order process occurs. We see that in the low-dose graphs (*L*_0_ < *R*_0_) the signature shape of Fig. 1 is no longer present. Instead, as *L*_0_ decreases, the ligand curves become increasingly bi-exponential, and condensed into an apparent mono-exponential decline. Still the terminal slope after the highest and the lowest dose are the same.

**Fig. 9:**
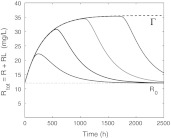
Simulated graphs of *R*_tot_(*t*) for the initial ligand concentrations *L*_0_ = 30, 100, 300, 900 mg/L and data from Table 3, together with the curve $\Upgamma$ (*dashed*) given by the analytic expression (20). Notice how, as *L*_0_ increases, the graph of *R*_tot_(*t*) follows $\Upgamma$ over a longer period of time

As the ligand doses decrease, the target profile becomes less affected in terms of intensity (depth) and duration below the baseline concentration. In fact, one can show that if *R*(0) = *R*_0_ and *RL*(0) = 0, then

11 $$ \frac{R_0}{\displaystyle{1+\frac{k_{\rm on}}{k_{\rm in}}\frac{L_0}{R_0}}} < R(t) < R_0\left(1+\frac{k_{\rm off}}{k_{\rm out}}\frac{L_0}{R_0}\right) \quad \hbox {for} \,0<t<\infty $$

The proof of this upper and lower bound is given in Appendix 3. It is immediately clear that when *L*_0_ → 0 the upper as well as the lower bound converges to *R*_0_. Therefore, we may conclude that for every *t* > 0,

12 $$ R(t) \to R_0 \quad \hbox {as} \, L_0 \to 0 $$

When we replace *R*(*t*) by *R*_0_ in the system (9) the resulting system is linear, involving only *L* and *RL*. This explains the bi-linear character of the log(*L*) versus time graphs (see also [17]).

### Mathematical analysis

We successively describe the dynamics in the four phases: *A*–*D*. Throughout we assume that the ligand has large affinity for the receptor, that the elimination rates are comparable, and that the bolus dose is not too small. Specifically we assume:

13 $$ {\bf A:}\,\varepsilon \stackrel{{\rm def}}{=} \frac{K_d}{R_0} \ll 1; \quad {\bf B:} \,\frac{k_{e(L)}}{k_{\rm off}},\,\frac{k_{e(RL)}}{k_{\rm off}},\,\frac{k_{\rm out}}{k_{\rm off}} <M; \quad {\bf C:}\, L_0 > R_0 $$

where *M* is a constant which is not too large, i.e., $\varepsilon M \ll 1.$ These three assumptions were inspired by the parameter values given in Table 3 and the initial values of *L* and *R*. We also mention a similar model used for the study of Interferon-β 1a in humans [23] fitted with comparable parameter values.

#### *Phase A *

Ligand, receptor and receptor–ligand complex quickly reach *Plateau values* (${\overline L}, {\overline R}, {\overline{RL}}$) (see the right graph of Fig. 5). Since *L*_0_ > *R*_0_ by Assumption B, the supply of free receptor is quickly exhausted, so that these plateau values are approximately given by

14 $$ {\overline L}=L_0-R_0, \quad {\overline R}=0, \quad {\overline{RL}} = R_0 $$

Note that this is confirmed by the initial portion of the graph of *L*(*t*) shown in Fig. 5: we see that *L* drops by approximately *R*_0_ over a time span of about 0.04 h. In Appendix 2 we give details of the dynamics in this initial phase and the approach to the plateau values. We show that it takes place over a time interval (0, *T*_1_), where^1^

15 $$ T_1 = O\left(\frac{1}{k_{\rm on}(L_0-R_0)}\right) \quad \hbox {as} \, k_{\rm on} \to \infty $$

When *L*_0_ = 900 mg/L this yields a half-life *t*_1/2_ of about 0.01 h, in agreement with what is shown in Fig. 5. For a detailed study of *Phase A* we refer to [17] and to Aston et al. [24].

#### *Phase B *

Over the subsequent time span when *L*(*t*) ≫ *K*_*d*_, say over the interval *T*_1_ < *t* < *T*_2_, the three compounds are in quasi-equilibrium. This means that *R*, *RL* and *R*_tot_ = *R* + *RL* are—approximately—related to *L* through the expressions:

16 $$ RL = R_{\rm tot}\frac{L}{L+K_d} \quad \hbox {and} \quad R = R_{\rm tot}\frac{K_d}{L+K_d} \quad \hbox {for} \, t>T_1 $$

Since in this phase *L*(*t*) ≫ *K*_*d*_, they may actually be approximated by the simpler expressions

17 $$ RL = R_{\rm tot} \quad \hbox {and} \quad R = 0 \quad \hbox {for} \, T_1 < t < T_2 $$

With these equalities, the system (4) can be reduced to the simpler form

18 $$ \left\{ \begin{array}{l} \frac{dL_{\rm tot}}{dt} = - k_{e(L)}L - k_{e(RL)} R_{\rm tot} \\ \frac{dR_{\rm tot}}{dt} = k_{\rm in} - k_{e(RL)} R_{\rm tot}\\ \end{array} \right. \quad T_1<t<T_2 $$

Note that for *R*_tot_ we have obtained a simple indirect response equation (see also [9]).

#### *Phase C* 

When *L*(*t*) = *O*(*K*_*d*_), say over the interval *T*_2_ < *t* < *T*_3_, the approximation (17) is no longer valid. Applying a scaling argument appropriate for this regime, we show in Appendix 5 that to good approximation

19 $$ RL = R_{\rm tot}\frac{L}{L+K_d} \quad \hbox {and} \quad R = R_{\rm tot}\frac{K_d}{L+K_d} \quad \hbox {for} \quad  T_2< t <T_3 $$

so that in this phase the *rapid binding assumption* is approximately satisfied [18].

#### *Phase D* 

When *L*(*t*) ≪ *K*_*d*_, i.e., beyond *T*_3_, the ligand concentration is so small that the dynamics is linear again.

The critical times *T*_1_, *T*_2_ and *T*_3_ provide a natural division of the dynamics in four phases: *A*, *B*, *C* and *D*, as was done in Fig. 1. In *Phase A* (0 < *t* < *T*_1_), ligand, receptor and complex reach quasi-equilibrium, in *Phase B* (*T*_1_ < *t* < *T*_2_), the bulk of the ligand is eliminated from the system while most of the receptor is bound to ligand and in quasi-equilibrium. *Phase C* (*T*_2_ < *t* < *T*_3_) is a nonlinear transitional phase in which *L* exhibits a steep drop, and finally, in *Phase D* ($T_3<t<\infty$) the three compounds converge linearly towards their baseline values.

### Receptor graphs: Phase B

In *Phase B*, which extends over the interval *T*_1_ < *t* < *T*_2_, the system (18) holds. Since the second equation only involves *R*_tot_ as a dependent variable, it can be solved explicitly to yield

$$ R_{\rm tot}(t) =R_* + \left\{R_{\rm tot}(T_1) - R_* \right\} e^{-k_{e(RL)}(t-T_1)} \quad \hbox {for} \, T_1<t<T_2 $$

Because *T*_1_ is small by the estimate (15), it follows from the system (4) and the initial conditions that *R*_tot_(*T*_1_) ≈ *R*_0_. Thus, to good approximation, we may put *T*_1_ = 0 and *R*_tot_(0) = *R*_0_, and so simplify the above expression for *R*_tot_(*t*) to

20 $$ R_{\rm tot}(t) =R_* + \left(R_0 - R_* \right)e^{-k_{e(RL)} t} \quad \hbox {for} \, 0<t<T_2 $$

where we recall from Eq. (10) that R_*_ = *k*_in_/*k*_*e*(*RL*)_. Plainly, if *T*_2_ were infinite, then

21 $$ R_{\rm tot}(t) \to R_* \quad \hbox {as} \, t \to \infty $$

We denote the graph of the function *R*_tot_(*t*) by $\Upgamma.$

In Fig. 9 we see how for the different drug doses, the simulations of *R*_tot_(*t*) follow the graph $\Upgamma$ up till some time, when they suddenly depart from $\Upgamma.$

#### *Remark*

We recall from the approximation (17) that *R*(*t*) ≈ 0 for *T*_1_ < *t* < *T*_2_ and hence that *RL*(*t*) ≈ *R*_tot_ in this phase of the dynamics, as we see confirmed in Fig. 6.

We conclude with a bound of the target pool. It is evident from the receptor graphs in Figs. 6, 7, 8 and 9 that for the data of the case study, the total receptor concentration *R*_tot_ is not constant. However, it remains bounded for all time and does not keep on growing. In fact it is possible to prove the boundedness of *R*_tot_ under very general conditions on the data, and actually obtain a sharp value for the upper bound. Specifically, we have

22 $$ R_{\rm tot}(t) \le \left\{ \begin{array}{ll} R_0 \quad &\hbox {if}\, k_{e(RL)} \ge k_{\rm out}\\ R_* \quad &\hbox {if}\, k_{e(RL)} \le k_{\rm out}\\\end{array}\right. \quad \hbox {for}\, t \ge 0. $$

The proof is given in Appendix 3.

### Ligand graphs: Phase B

Over the interval (*T*_1_,*T*_2_) in which it is assumed that *L* ≫ *K*_*d*_, we can use (17) to simplify the first equation from the system (18) as follows:

23 $$ \frac{d}{dt} \left( L + R_{\rm tot} \right) = - k_{e(L)}L - k_{e(RL)} R_{\rm tot} \quad \hbox {for} \, T_1<t<T_2 $$

Subtracting the second equation of (18) from (23) we obtain

24 $$ \frac{dL}{dt} = - k_{e(L)}L - k_{\rm in} \quad \hbox {for} \, T_1<t<T_2 $$

Equation (24), together with the initial value $L(T_1)={\overline L},$ can be solved explicitly. In Appendix 4 we show that the solution is given by

25 $$ L_{\rm approx}(t) = \left({\overline L} + \frac{k_{\rm in}}{k_{e(L)}}\right)e^{-k_{e(L)}t}- \frac{k_{\rm in}}{k_{e(L)}} \quad \hbox {for} \, 0<t<T_2 $$

where *T*_1_, being small, has been put equal to zero. We recall from Eq. (14) that ${\overline L} \approx L_0-R_0.$

**Fig. 10:**
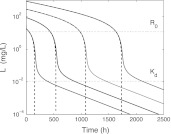
Graphs of *L* versus time on a semi-logarithmic scale for data as in Fig. 5. The *dashed curves* are the analytic approximations for the different drug doses, given by Eq. (25). Recall from Eq. (7) that *K*_*d*_ = 0.011 mg/L

In Fig. 10 we compare the numerically computed ligand versus time graphs *L*(*t*) with the analytic approximation *L*_approx_(*t*) given by the expression (25). It is evident that the two curves are very close until *L* has become so small that it is comparable to *K*_*d*_, i.e., until the end of Phase *B*, where the nonlinearity pitches in.

Since the function *L*_approx_(*t*) is decreasing, *L*_approx_(0) > 0 and *L*_approx_(*t*) → − *k*_in_/*k*_*e*(*L*)_ < 0 as $t \to \infty,$ it follows that there exists a unique time *T* ^*^ > 0 at which *L*_approx_(*t*) vanishes. We readily conclude from the definition of *L*_approx_(*t*) that *T* ^*^ is given by

26 $$ T^* = \frac{1}{k_{e(L)}}\log\left\{\frac{k_{e(L)}}{k_{\rm in}}(L_0-R_0) +1\right\} $$

#### *Remark*

It is clear from Eq. (24) that for larger values of *L*_0_, we may estimate *k*_*e*(*L*)_ from the slope of log(*L*) and in Fig. 10 we see that *T* ^*^ yields a good estimate for *T*_2_, the end of *Phase B*. Information about *k*_*e*(*L*)_ and *T* ^*^ combined yields an estimate for *k*_in_ from the expression

27 $$ k_{\rm in} = \frac{k_{e(L)}}{e^{k_{e(L)}T^*}-1}(L_0-R_0) $$

which can be derived from (26).

When *K*_*d*_ is very small, as is the case for the parameter values of Table 1, the approximation *L*_approx_(*t*) given by (25) is valid for most of the range of *L* and can therefore be used to obtain an estimate for the area under the ligand curve *AULC*. An elementary computation yields

28 $$ \begin{aligned} AULC(D) &= \int\limits_0^{\infty} L(t,L_0) dt \\ &\approx \int\limits_0^{T^*} L_{\rm approx}(t,L_0) dt \\ &= \frac{D}{k_{e(L)}V_c} \left\{1-\mu-{\kappa} \mu \log{\left(\frac{1+({\kappa}-1) \mu}{{\kappa} \mu}\right)}\right\}, \quad \mu = \frac{R_0}{L_0}, \quad {\kappa} = \frac{k_{\rm out}}{k_{e(L)}}\\ \end{aligned} $$

Details are given in Appendix 4. This expression for *AUCL* yields for the clearance

29 $$ CL(D) = \frac{D}{AULC(D)} \approx k_{e(L)}V_c \left\{1 -\mu-{\kappa} \mu \log{\left(\frac{1+({\kappa}-1)\mu}{{\kappa} \mu}\right)}\right\}^{-1} $$

It is interesting to compare these approximate expressions for *AUCL* and *CL* with the corresponding expressions for mono-exponential ligand elimination. They are, respectively, *D*/(*k*_*e*(*L*)_ *V*_*c*_) and *k*_*e*(*L*)_ *V*_*c*_. Thus we see that they are related by a factor which only depends on two *dimensionless* critical numbers:

- (i) the ratio μ of *R*_0_ and *L*_0_ and
- (ii) the ratio κ of the direct elimination rates of receptor, *k*_out_, and ligand, *k*_*e*(*L*)_.

Since μ→ 0 as $L_0 \to \infty,$ we conclude from (28) and (29) that

$$ AULC(D) \sim \frac{D}{k_{e(L)}V_c} \quad \hbox {and} \quad CL(D) \to k_{e(L)}V_c \quad \hbox {as}\, D \to \infty $$

### Ligand and receptor graphs: Phases C and D

In *Phase C* the ligand concentration *L* has become comparable to *K*_*d*_ and, as is shown in Appendix 5, we have to good approximation

30 $$ L \cdot R = K_d \cdot RL \quad \hbox {and} \quad RL = R_{\rm tot}\frac{L}{L + K_d} $$

This suggests using a different scaling of *L*. For definiteness we assume that *Phase C* comprises the time interval in which *L* drops from 10 × *K*_*d*_ to 0.1 × *K*_*d*_, and denote the times that *L*(*t*) reaches 10 × *K*_*d*_ and 0.1 × *K*_*d*_ by, respectively, *T*_2_ and *T*_3_. Thus, *L*(*T*_2_) = 10 × *K*_*d*_ and *L*(*T*_3_) = 0.1 × *K*_*d*_.

In *Phase C* we use the approximation (30) in the ligand conservation law in (4):

31 $$ \frac{d}{dt}\left(L+R_{\rm tot}\frac{L}{L + K_d}\right) = -k_{e(L)}L - k_{e(RL)}R_{\rm tot}\frac{L}{L + K_d} $$

We now introduce *K*_*d*_, which—like *L*, *R* and *RL*, has the dimension of a concentration—as a reference variable for *L*, and introduce the dimensionless variables

32 $$ u(t) = \frac{L(t)}{K_d} \quad \hbox {and} \quad v(t) = \frac{R_{\rm tot}(t)}{R_0} $$

Using these variables in Eq. (31) we obtain

$$ \frac{d}{dt}\left( \varepsilon u + v\frac{u}{u + 1}\right) = - k_{e(L)} \varepsilon u - k_{e(RL)}v\frac{u}{u + 1}, \quad \varepsilon = \frac{K_d}{R_0} $$

Since $\varepsilon \ll 1,$ and *v* = *O*(1) (cf. Fig. 6), we may neglect the term $\varepsilon u$ in the left- and the right-hand side of this equation and so obtain

33 $$ \frac{d}{dt}\left(v\frac{u}{u + 1}\right) = - k_{e(RL)}v\frac{u}{u + 1} $$

In the simulations shown in Figs. 5 and 6 we see that in *Phase C*, *L* drops rapidly from *O*(10 × *K*_*d*_) to *O*(0.1 × *K*_*d*_), i.e., by a factor 100, whilst *R*_tot_ stays relatively close to R_*_ and changes by no more than a factor 1/7 ≈ 0.15. This suggests making the following assumption:

#### **Assumption**

*R*_tot_(*t*) ≈ R_*_ or *v*(*t*) ≈ R_*_/*R*_0_ in *Phase C*.

Thanks to this assumption we may view *v* as a constant, which we may divide out and thus eliminate from the equation. We end up with a simple nonlinear equation for *u*, which is valid in *Phase C*:

34 $$ \frac{du}{dt} = - k_{e(RL)} u(u + 1) \quad \hbox {for}\, T_2 < t < T_3 $$

Since $L(T_2)=10 \cdot K_d,$ it follows that *u*(*T*_2_) = 10. Equation (34) can be solved explicitly and we find for its solution:

35 $$ u(t) = \frac{A e^{-k_{e(RL)}(t-T_2)}}{1-Ae^{-k_{e(RL)}(t-T_2)}} \quad \hbox {for} \, t \ge T_2, \quad A=\frac{u(T_2)}{1+u(T_2)}= \frac{10}{11} = 0.91 $$

Returning to the original variables we obtain for the large time behaviour

36 $$ \log\{L(t)\} \sim \log(K_d) + \log(A) - k_{e(RL)}(t-T_2) \quad \hbox {as} \, t \to \infty $$

The asymptotic expression (36) yields estimates for

- (i) the terminal slope λ_*z*_^TMDD^ (*k*_*e*(*RL*)_);
- (ii) the *intercept* of the asymptote of log{*L*(*t*)} of the ligand graph in the terminal *Phase D* with the vertical line {*t* = *T*_2_}.

The approximate identities in (30) imply that in *Phases C* and *D*, when *L* = *O*(*K*_*d*_), we have to good approximation

37 $$ \frac{dRL}{dt} = - k_{e(RL)}RL $$

so that

38 $$ RL(t) \approx R_* e^{ - k_{e(RL)}t} $$

This is consistent with the value of λ_*z*_ found for *L*(*t*).

We also find that to good approximation

39 $$ \frac{dR}{dt} = k_{\rm in}- k_{\rm out}R $$

so that

40 $$ R(t) \approx R_0 (1- e^{-k_{\rm out}t}) $$

This confirms what we see in Fig. 7: that for *t* > *T*_2_ the receptor concentration *R*(*t*) tends to *R*_0_ in a bi-exponential manner, in contrast to the way *RL*(*t*) tends to zero, which is mono-exponential.

For completeness we also compute the terminal slope by means of a standard analysis of the full TMDD system. This is done in Appendix 7. It is found that for the parameter values in Table 3, the terminal slope λ_*z*_ of all the compounds, is given—to good approximation—by λ_*z*_^TMDD^ = *k*_*e*(*RL*)_. This confirms the limit in (36) and the exponent in (38).

### Comparison with Michaelis–Menten kinetics

In many studies involving TMDD, models are employed that combine linear and saturable Michaelis–Menten type elimination (e.g. see [14]) of the form

41 $$ \frac{dL}{dt} = - k L - V_{\rm max} \frac{L}{L+K_M} $$

in which *k*, *V*_max_ and *K*_*M*_ are empirical parameters. The underlying assumption is that the MM-term can replace the combined first and second order processes of buildup and elimination via the complex in the TMDD model within a certain ligand concentration range.

In light of (24), fitting the data for large values of the ligand concentration would yield *k* = *k*_*e*(*L*)_ and *V*_max_ = *k*_in_. Putting *K*_*M*_ = *K*_*d*_, Eq. (41) then becomes

42 $$ \frac{dL}{dt} = - k_{e(L)}L - k_{\rm in} \frac{L}{L+K_d} $$

Alternatively, we can take (31) as point of departure. Following [15] we assume that *R*_tot_ *K*_*d*_ ≪ (*K*_*d*_ + *L*)^2^. Then the left hand side of (31) reduces to *dL*/*dt*. Assuming that *R*_tot_ ≈ R_*_ and remembering that *k*_*e*(*RL*)_ R_*_ = *k*_in_, we may replace the factor *k*_(*e*(*RL*)_ *R*_tot_ in the right hand side of (31) by *k*_in_ and so arrive at (42).

Fitting to data of low ligand concentrations (*L* ≪ *K*_*d*_), Eq. (42) reduces to the linear equation

43 $$ \frac{dL}{dt} = - \left(k_{e(L)} +\frac{k_{\rm in}}{K_d}\right) L $$

which yields a terminal slope λ_*z*_^MM^ given by

44 $$ \lambda_z^{\rm MM} = k_{e(L)} +\frac{k_{\rm in}}{K_d} $$

This terminal slope is quite different from the value λ_*z*_^TMDD^ obtained in (36) and (38). For the parameter values of Table 3, we find that λ_*z*_^MM^ ≫ λ_*z*_^TMDD^.

Thus, the TMDD-model and the Michaelis–Menten (MM)-model exhibit very different terminal slopes, unless one also includes a non-specific peripheral volume distribution term in the MM-model.

**Fig. 11:**
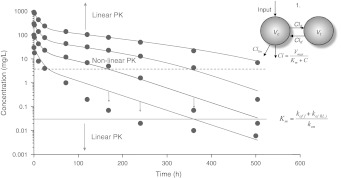
Fitting the 2-compartment Michaelis–Menten model (45) to the data of Fig. 3 which are represented by the *dots*. The *drawn curves* are predictions of the Michaelis–Menten model for the parameter values listed in Table 4. The *dashed line* in the middle of the plot indicates the estimated value of *K*_*M*_. Notice how far away it is from the original value of *K*_*m*_—marked by the *thin drawn line*—which was estimated by the TMDD model

**Michaelis–Menten model with peripheral compartment**. Adding a peripheral compartment to the MM-model makes it possible to capture the slow terminal elimination that is typically seen in TMDD data. Figure 11 shows the regression of a 2-compartment model with parallel linear (*Cl*_(*L*)_) and Michaelis–Menten (*Cl* = *V*_max_/(*L*_*p*_ + *K*_*M*_)) elimination:

45 $$ \left \{ \begin{array}{ll} V_c\frac{dL_p}{dt} = - Cl_{(L)} L_p - Cl_{d}(L_p-L_t) - V_{\rm max}\frac{L_p}{L_p +K_M} \\ V_t\frac{dL_t}{dt} = Cl_{d}(L_p-L_t) \\ \end{array}\right. $$

Fitting this model to the data shown in Fig. 3 results in the parameter estimates which, together with their precision (CV%), are given in Table 4.

The reduced model mimics the concentration–time data for the two highest doses reasonably well, whereas the two lower doses display systematic deviations between observed and predicted data.

Since the reduced model has two parallel elimination pathways (linear and nonlinear) it has the intrinsic capacity of exhibiting linear first-order kinetics at low and at high concentrations. In the concentration-range in between it behaves nonlinearly. For higher concentrations the MM-route is saturated and the linear elimination pathway dominates so that the system behaves linearly.

However, the typical concentration–time pattern for ligand seen in a true TMDD system (cf. Figs. 5, 8, 10), cannot be fully described by the parallel linear- and MM-elimination model. The reduced model displays typical bi-exponential decline (which is expected from a two-compartment model) at lower concentrations. That is generally not the case with the full TMDD model.

A clear distinction of the two models occurs initially, immediately after dosing (*Phase A*), when the second-order reaction between ligand and circulating target forms the complex. This process cannot be captured by the reduced model, which may cause biased estimates (too large) of the central volume.

In Table 5 we summarise these results. It shows that the MM-models (41) and (45) may be fitted successfully to the first part of the ligand versus time graph, although they miss the initial drop in *Phase A*. They catch the first part of *Phase C*, but the first model fails to catch the second part, where the graph joins up with the terminal *Phase D*. The second MM-model is an improvement, but still shows significant deviations for lower ligand-concentrations.

**Table 4:** Parameter estimated from fitting the Michaelis–Menten model (Eq. (45)) to the data shown in Fig. 11. As in the Case Study, *V*_*c*_ is fixed

Symbol	Unit	Value	CV%
*V*_*c*_	L/kg	0.05	–
*V*_*t*_	L/kg	0.1	10
*Cl*_*d*_	(L/kg)/h	0.00307	20
*Cl*_(*L*)_	(L/kg)/h	0.00090	10
*V*_max_	mg/h	0.0146	40
*K*_*M*_	mg/L	3.68	50

**Table 5:** Phases that can be explained by the two MM-models and the TMDD-model

Phase	MM-model (41)	MM-model (45)	TMDD-model (9)
*A*	−	−	+
*B*	+	+	+
*C*	+/−	+	+
*D*	−	−	+

In this table, a plus (+) means that the corresponding phase can be adequately explained, whilst a minus (−) means that it cannot.

## Constant rate drug infusion

We assume that the drug is administered through a constant-rate infusion over a finite period of time, and we are interested in elucidating which parameters are critical in determining the time to steady state, the extent of steady state ligand, target and ligand–target complex concentrations and the dynamics after washout. Assuming that the infusion rate reaches its constant value *k*_*f*_ in a negligible amount of time and that washout at time *t*_washout_ is also instantaneous, we consider the following variant of the system (1) :

46 $$  \left\{ \begin{array}{l} \frac{dL}{dt} = k_fH(t_{\rm washout}-t) -k_{\rm on} L \cdot R + k_{\rm off} RL - k_{e(L)}L \\ \frac{dR}{dt} = k_{\rm in} - k_{\rm out} R -k_{\rm on} L \cdot R + k_{\rm off} RL \\ \frac{dRL}{dt} = k_{\rm on} L \cdot R - (k_{\rm off}+ k_{e(RL)})RL \\\end{array}\right. $$

in which *H*(*t*) denotes the *Heaviside function*: *H*(*t*) = 0 if *t* < 0 and *H*(*t*) = 1 if *t* > 0. Thus, *H*(*t*_washout_ − *t*) = 1 if *t* < *t*_washout_ and *H*(*t*_washout_ − *t*) = 0 if *t* > *t*_washout_. We assume that initially there is no ligand in the system, i.e., *L*_0_ = 0.

When the infusion lasts long enough, i.e., when *t*_washout_ is large enough, the concentrations will converge towards their steady state values *L*_ss_, *R*_ss_ and *RL*_ss_. Then, at washout, they will return to their pre-infusion values: *L* = 0, *R* = *R*_0_ and *RL* = 0.

We first derive expressions for the steady state values. Then we carry out a series of simulations subject to the same assumptions as those made in (13), except that we replace Assumption C by

13* $$ {\bf C^*:}\, L_{\rm ss}(k_f) > R_0$$

As we shall see, *L*_ss_ is an increasing function of *k*_*f*_, so that we require here that *k*_*f*_ does not drop below a threshold value for which *L*_ss_(*k*_*f*_) = *R*_0_. We discuss features of the dynamics exhibited in these simulations, especially the time to steady state after onset of infusion, and after washout.

### Steady state concentrations of *L*, *R* and *RL*

For the steady state concentrations *L*_ss_, *R*_ss_ and *RL*_ss_ of the system (46) we find the following expressions. For the ligand–receptor concentration we obtain

47 $$ RL_{\rm ss}=\frac{1}{2k_{e(RL)}}\left\{k_f+ k_{\rm in} + q-\sqrt{(k_f+ k_{\rm in} + q)^2 - 4k_f k_{\rm in}} \right\}, $$

in which *q* = (*k*_*e*(*L*)_/*k*_*e*(*RL*)_)*k*_out_ *K*_*m*_. In light of the conservation laws for ligand and target, we then obtain for the ligand

48 $$ L_{\rm ss}= \frac{1}{k_{e(L)}} (k_f- k_{e(RL)}RL_{\rm ss}) $$

and for the target,

49 $$ R_{\rm ss}= \frac{1}{k_{\rm out}} (k_{\rm in}- k_{e(RL)}RL_{\rm ss}) $$

The expressions (47)–(49) are derived in Appendix 6.

The formula (48) for the ligand shows that *L*_ss_ will be smaller than expected from the ratio of ligand infusion rate-to-clearance (*In*_*L*_/*Cl*_(*L*)_ = *k*_*f*_/*k*_*e*(*L*)_), due to the removal of ligand as part of the complex *RL*_ss_. The same reasoning may be used to explain why the circulating target concentration *R*_ss_ given by (49) is smaller than the baseline concentration *R*_0_ = *k*_in_/*k*_out_. Due to the removal of target by means of the complex, the target concentration *R*_ss_ will drop further as the infusion rate increases and *RL*_ss_ increases accordingly (cf. Eq. (49)).

**Fig. 12:**
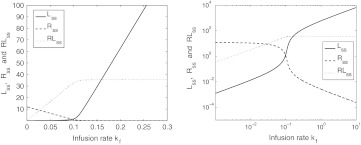
The steady state concentrations *L*_ss_, *R*_ss_ and *RL*_ss_ graphed versus the infusion rate *k*_*f*_, on a linear scale (*left*) and on a log-log scale (*right*) for parameter values taken from Table 3

Figure 12 shows graphs of *L*_ss_, *R*_ss_ and *RL*_ss_ as functions of the infusion rate *k*_*f*_ for the parameter values of Table 3. Note that at large infusion rates, *L*_ss_ increases approximately linearly, except for a downward shift. Indeed, this is confirmed analytically: by expanding the expression (48) for large values of *k*_*f*_, we obtain

50 $$ L_{\rm ss}(k_f) \sim \frac{1}{k_{e(L)}}(k_f - k_{\rm in}) \quad \hbox {as} \, k_f \to \infty $$

The reason is that for large infusions, elimination of ligand occurs primarily via the extra-target elimination route (*k*_*e*(*L*)_). For small infusion rates the removal of ligand is also seen to be first order, but clearly at a much lower rate.

In Fig. 12 the receptor concentration *R*_ss_ is seen to *decrease* and the complex concentration *RL*_ss_ is seen to *increase* as *k*_*f*_ increases. However, it is interesting to observe that *RL*_ss_ reaches an upper bound in spite of increasing levels of *k*_*f*_. This is also confirmed analytically: letting *k*_*f*_ tend to infinity in (47) and (49) we obtain the limits

51 $$ R_{\rm ss}(k_f) \to 0 \quad \hbox {and} \quad RL_{\rm ss}(k_f) \to R_* \quad \hbox {as} \, k_f \to \infty $$

Thus, the upper bound is found to be R_*_ = *k*_in_/*k*_*e*(*RL*)_.

It is interesting to note that when plotted on a logarithmic scale, the elimination of target mirrors the elimination of ligand for larger values of the infusion rate (cf. Fig. 12 on the right). This can be understood from the relation

52 $$ k_{\rm on} L_{\rm ss} \cdot R_{\rm ss} - (k_{\rm off}+ k_{e(RL)})RL_{\rm ss}=0 $$

obtained from (46). When we divide Eq. (52) by *k*_on_ and take the logarithm, we obtain

$$ \log(L_{\rm ss}) + \log(R_{\rm ss}) = \log(K_m) + \log(RL_{\rm ss}) $$

For larger values of *k*_*f*_ we have *RL*_ss_ ≈ R_*_ (cf. (51)), and hence

53 $$ \log(L_{\rm ss}) + \log(R_{\rm ss}) \approx \log(K_m) + \log(R_{*}) $$

which establishes the symmetry which is evident in the graphs for *L*_ss_ and *R*_ss_ shown on the right in Fig. 12.

Using the parameter values in Table 3, we obtain 1/*k*_*e*(*L*)_ = 667, *k*_in_/*k*_*e*(*L*)_ = 73, R_*_ = *k*_in_/*k*_*e*(*RL*)_ = 36.7 and log(*K*_*m*_) + log(R_*_) = 0.45. We see that these values are confirmed by the numerically obtained graphs shown in Fig. 12.

We note that the expressions (47)–(49) show that the steady state concentrations do not depend on the on- and off rates *k*_on_ and *k*_off_ individually, but only as part of the constant *K*_*m*_.

### Simulations

**Fig. 13:**
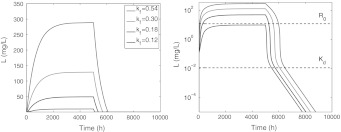
The ligand concentration *L* graphed versus time on a linear scale (*left*) and on a semi-logarithmic scale (*right*) for the infusion rates *k*_*f*_ = 0.12, 0.18, 0.30 and 0.54 (mg/L)/h and *t*_washout_ = 5000 h. The parameter values are taken from Table 3

**Fig. 14:**
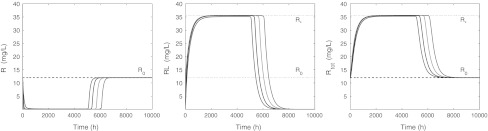
Concentration profiles of *R*, *RL* and *R*_tot_ versus time caused by a constant rate infusion of 5000 h and infusion rates of *k*_*f*_ = 0.12, 0.18, 0.30 and 0.54 (mg/L)/h. The parameter values are taken from Table 3. Note that the time to full depletion of target *R* decreases as the infusion rate *k*_*f*_ of ligand increases

**Fig. 15:**
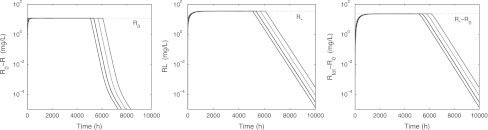
Graphs of *R*_0_ − *R*, *RL* and *R*_tot_ − *R*_0_ versus time when *k*_*f*_= 0.12, 0.18, 0.30 and 0.54 (mg/L)/h. The parameter values are taken from Table 3. Note that the convergence of target *R* to *R*_0_ is bi-exponential and that the decline of complex *RL* to zero, and the convergence of total target *R*_tot_ to *R*_0_ are mono-exponential

We show simulations of concentration versus time graphs of the system (46) when drug is supplied through a constant-rate infusion over a period of 5000 h. Four infusion rates are considered: *k*_*f*_ = 0.12, 0.18, 0.30 and 0.54 (mg/L)/h. In Fig. 13 we show ligand graphs, on a linear and on a semi-logarithmic scale, and in Figs. 14 and 15 we show graphs of the concentration of *R*, *RL* and *R*_tot_, first on a linear scale and then also on a logarithmic scale. In each of these figures we include the build-up phase as well as the washout phase.

In Fig. 13 we see that for larger values of *k*_*f*_ the time-to-steady state of the ligand *L* is more or less independent of the infusion rate. The amounts of ligand are now so large that the receptor is quickly saturated and ceases to play an important role in the dynamics. What remains is the linear clearance of ligand, so that the ligand dynamics is described to good approximation by the equation

54 $$ \frac{dL}{dt} = k_f - k_{e(L)}L - k_{\rm in} $$

which can be solved explicitly. Evidently

55 $$ L(t) \to \frac{1}{k_{e(L)}}(k_f - k_{\rm in}) \quad \hbox {as} \, t \to \infty $$

which is consistent with (50) and

56 $$ t_{1/2} = \frac{\log(2)}{k_{e(L)}} $$

This results in a time to steady-state of 3–4 × *t*_1/2_ is ≈1850 h for the parameter values given in Table 3.

The washout dynamics is very similar to the dynamics after a bolus dose, as described before: *Phase A* (0,*T*_1_) now covers the infusion period so that *T*_1_ coincides here with the time of washout. *Phases B–D* are plainly evident in the post-washout dynamics.

The dynamics of the receptor *R* and the receptor–ligand complex *RL* are shown in Figs. 14 and 15. As in *Phase A* in the bolus administration, the pre-dose receptor pool (*R*_0_) quickly binds to the ligand. We see that the speed of receptor depletion increases with increasing infusion rate *k*_*f*_, consistent with the half-life estimate (15) after a bolus administration.

In due course, additional receptor is formed, albeit more slowly—and binds immediately to the ligand—resulting in an increase of *RL* as was observed after a bolus dose (see also Fig. 6). The dynamics is very similar—compare Eq. (20)—and

57 $$ t_{1/2} = \frac{\log(2)}{k_{e(RL)}} $$

This results in a time-to-steady-state of 3–4 × *t*_1/2_ is ≈924 h (cf. Table 3), which we see confirmed in Figs. 14 and 15. Eventually *RL*(*t*) levels off at the steady state value *RL*_ss_, which is close to R_*_ for the larger infusion rates (cf. Eq. (51)).

After washout, when *k*_*f*_ is large, *R*(*t*) ≈ 0 and *RL*(*t*) ≈ R_*_ for a while before they abruptly return to their baseline values. It is evident from Fig. 15 that, as in Fig. 7, initially the slope of log(*R*_0_ − *R*) is steeper than that of log(*RL*). This is in agreement with the analysis presented in Appendix 5, where it is shown that over that period the half-lives of *R*(*t*) − *R*_0_ and *RL*(*t*) are, respectively, *O*(1/*k*_out_) and *O*(1/*k*_*e*(*RL*_).

## Discussion and conclusion

We have shown how the concentration profile of ligand, receptor and ligand–receptor complex in the TMDD model can be divided into four different phases and how for each of these phases closed-form approximations can be derived. Inspired by a specific case study, the following assumptions were made about the parameter values (see also (13) and (13*)):

58 $$ \varepsilon = \frac{K_d}{R_0}\ll 1,\quad \alpha = \frac{k_{e(L)}}{k_{\rm off}}<M,\quad\beta = \frac{k_{\rm out}}{k_{\rm off}}<M,\quad \gamma= \frac{k_{e(RL)}}{k_{\rm off}} < M $$

for some moderate constant *M* > 0, and *L*_0_ > *R*_0_.

When ligand is administered through a bolus dose, *L*_0_ > *R*_0_, and the conditions in (58) are satisfied, four phases can be distinguished in the ligand elimination graph: a brief initial *Phase* *A*, a slow linear *Phase* *B*, a rapid nonlinear *Phase* *C* and then again a slow linear terminal *Phase* *D* (cf. Fig. 1). Thanks to accurate analytical approximations for these four phases as shown in the Eqs. (14) for *Phase A*, (20), (25)–(27) for *Phase B*, (36) for *Phase C* and (38) and (40) for *Phase D*, we may extract information about the model parameters.

In Table 6 we list the parameter values which play a central role in the different phases of TMDD graphs. In *Phase A* : the drop (*R*_0_) and the duration (*O*(1/(*k*_on_ *L*_0_))), in *Phase B* : the slope (*k*_*e*(*L*)_) and the receptor input (*k*_in_), in *Phase C* : the depth (*K*_*d*_) and in *Phase D* : the terminal slope (*k*_*e*(*RL*)_). In brackets we have included the parameters which can be estimated when results from earlier phases are used. Thus, since *Phase A* yields an estimate for *R*_0_ = *k*_in_/*k*_out_ and *Phase B* an estimate for *k*_in_, an estimate for *k*_out_ follows.

**Table 6:** Information contained in the four phases

Phase	TMDD-model (9)
*A*	*R*_0_ and *k*_on_
*B*	*k*_*e*(*L*)_, *k*_in_ (*k*_out_)
*C*	*K*_*d*_ (*k*_off_)
*D*	*k*_*e*(*RL*)_ (*K*_*m*_)

It should be noted though that estimating *R*_0_ may be difficult, since often there are no data for the first phase because it is over very quickly.

**Fig. 16:**
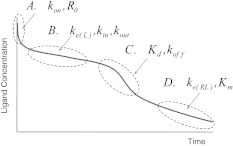
Schematic representation of how the parameters may be derived from properties of the four phases. In *Phase A* ligand binds to the receptor (*k*_on_), during *Phase B* ligand is primarily eliminated directly (*k*_*e*(*L*)_); time of termination yields information about *k*_in_. In *Phase C* the saturation term is important (*K*_*d*_), and in *Phase D* ligand elimination proceeds mainly though the receptor (*k*_*e*(*RL*)_)

In Fig. 16 we summarise these results and show in the schematic ligand versus time graph (recall Fig. 1) the parameters which may be estimated from the different phases.

The four phases identified in the ligand elimination graph after a bolus administration are reflected in the structure of the receptor versus time graphs (receptor, receptor–ligand complex, and total amount of receptor). During *Phase* *A* the receptor pool is quickly depleted, and it remains so during *Phase* *B*. Then, during the *Phases* *C* and *D* it climbs back to the terminal baseline level *R*_0_.

The analytic approximations obtained for *L*, *R* and *R*_tot_ may be used to verify the validity of the assumptions which underpin different approximations to the full TMDD model when the Assumptions A, B and C (or C^*^) regarding the parameters are satisfied.

**Fig. 17:**
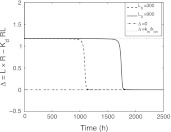
Evolution of the quantity $\Updelta = L \cdot R - K_d RL$ with time for two initial doses *L*_0_ = 300 and 900. Parameter values are taken from Table 1. Note the agreement with the analytical predictions made above for *Phases B, C* and *D*

1. The *rapid binding model* [6, 18], in which it is assumed that
  59 $$ L \cdot R = K_d RL $$
  where *K*_*d*_ is defined in (7). In *Phase B*, which is characterised by *R*(*t*) ≈ 0, we have *dR*/*dt* ≈ 0 so that, according to the second equation of the system (9), we have approximately
  60 $$ \Updelta \mathop{=}\limits^{{\rm def}} L \cdot R - K_d RL =\frac{k_{\rm in}}{k_{\rm on}} $$
  which disagrees with (59).
  In contrast, in *Phases C and D* the identity (59) is satisfied according to the results established in Appendix 5 (cf. (102)), and Appendix 7 where it was shown that λ_*z*_ = *k*_*e*(*RL*)_.
  In Fig. 17 we show how the quantity $\Updelta = L \cdot R - K_d RL$ varies with time and how $\Updelta$ rapidly jumps from *k*_in_/*k*_on_ down to zero at the transition of *Phases B and C*.
2. The *quasi-steady-state model* [7, 18] in which it is assumed that
  61 $$ L \cdot R = K_m RL $$
  where *K*_*m*_ is defined in (8). Evidently, this assumption is not valid during *Phases C and D*, but it is during that part of *Phase B* in which *RL*(*t*) ≈ R_*_. In that interval *dRL*/*dt* ≈ 0 (cf. Fig. 6) and hence, by the third equation of the system (1), condition (61) is approximately satisfied.

Anchored on the data of the Case Study, the analysis in this paper is based on the Assumptions A, B and C (or C^*^). The question arises whether the characteristic features of the ligand elimination curves, such as shown in Fig. 1, are still present when these assumptions are not met.

In general, the behaviour of nonlinear systems such as (1) is very sensitive to the values of the parameters and initial data involved. However, a number of features of the ligand versus time graphs is quite robust in that they may survive if e.g. Assumption A is not satisfied and *K*_*d*_ and *R*_0_ are comparable. Thus, the estimate (15) for *T*_1_ suggests that the initial *Phase A* will remain short relative to typical times over which the other processes develop when *K*_*d*_/*L*_0_ is small. We refer to [17] and [18] for a detailed analysis of this situation.

The approximate expressions for *L* and *R*_tot_ in *Phase B* (Eqs. (25) and (20)) are still valid provided that *R*(*t*) ≈ 0. This will still be the case when Assumption A is replaced by *K*_*d*_ ≪ *L*_0_ [17, 18].

In contrast, the analysis of the dynamics in *Phase C* that is carried out in Appendix 5 depends critically on Assumption A. It will be interesting to study the dynamics beyond *Phase B* when Assumption A does not hold, as it will be interesting to see how the value of α, β and γ affects the dynamics.

We have selected a set of data (ligand and circulating target and complex) with low experimental variability, concentration–time courses at four ligand doses given as bolus injections, and well-spaced data in time that captures the necessary phases and shapes of a typical TMDD system. Based on this approach and the mathematical/analytical analysis, we can draw conclusions about the identifiability of the model parameters and appropriate system. When data are less precise and information rich, or, when target and/or complex are less accessible, the a priori expectations of parameter accuracy and precision will be lower.

## Appendix 1: Symbols and definitions

### Concentrations and related quantities

*L*: Ligand concentration (mg/L)
*R*: Receptor concentration (mg/L)
*RL*: Receptor–ligand concentration (mg/L)
*L*_tot_: Total ligand concentration (*L* + *RL*) (mg/L)
*R*_tot_: Total receptor concentration (*R* + *RL*) (mg/L)
*L*_0_: Initial ligand concentration (mg/L)
*R*_0_: Initial receptor concentration (mg/L)
*R*_*_: Intermediate steady state receptor concentration (mg/L)
${\overline L}$: Plateau value of the ligand concentration (mg/L)
${\overline R}$: Plateau value of the receptor concentration (mg/L)
${\overline{RL}}$: Plateau value of the receptor–ligand concentration (mg/L)
*L*_ss_: Steady state ligand concentration (mg/L)
*R*_ss_: Steady state receptor concentration (mg/L)
*RL*_ss_: Steady state receptor–ligand concentration (mg/L)
*L*_approx_: Approximation of the ligand concentration (mg/L)
*AULC*: Area Under the Ligand Concentration curve (mg/h/L)
*CL*: Apparent clearance of ligand (L/h)
λ_*z*_: terminal slope (h^−1^)

### Parameters in the model

*In*_*L*_: Infusion rate of ligand (mg/h)
*V*_*c*_: Volume of the central compartment (L)
*V*_*t*_: Volume of the peripheral compartment (L)
*Cl*_*d*_: Inter-compartmental distribution (L/h)
*Cl*_(*L*)_: Direct ligand elimination (L/h)
*k*_*f*_: *In*_*L*_/*V*_*c*_ (mg/L/h)
*k*_*e*(*L*)_: First-order elimination rate constant of ligand (h^−1^)
*k*_in_: Turnover rate of receptor (mg/h)
*k*_out_: Fractional turnover rate of receptor (h^−1^)
*k*_on_: Second order association rate of ligand to receptor ((mg/L)^−1^/h)
*k*_off_: First-order dissociation rate of receptor–ligand complex (h^−1^)
*k*_*e*(*RL*)_: First-order elimination rate of receptor–ligand complex (h^−1^)
*K*_*d*_: *k*_off_/*k*_on_ (mg/L)
*K*_*m*_: (*k*_off_ + *k*_*e*(*RL*)_)/*k*_on_ (mg/L)
*K*_*M*_: Michaelis–Menten constant in Eq. (45) (mg/L)
*V*_max_: Maximum elimination rate in Eq. (45) (mg/kg/h)
*T*_1_, *T*_2_, *T*_3_: Times where the ligand versus time curve changes qualitatively (h)
*T*^*^: Time when *L*_approx_(*t*) vanishes (cf. Eqs. (25) and (26)) (h)

### Dimensionless quantities

*x*, *y*, *z*: *L*/*L*_0_, *R*/*R*_0_, *RL*/*R*_0_; dimensionless concentrations
$${\overline x}, {\overline y}, {\overline z}$$: $${\overline L}/L_0, {\overline R}/R_0, {\overline{RL}}/R_0; $$ dimensionless concentrations
*u*, *v*, *w*: *L*/*K*_*d*_, *R*/*R*_0_, *RL*/*R*_0_; dimensionless concentrations
τ: *k*_on_ *R*_0_ *t*; dimensionless time
ɛ: *K*_*d*_/*R*_0_
κ: *k*_out_/*k*_*e*(*L*)_
μ: *R*_0_/*L*_0_ or *R*_0_/*L*_ss_
α: *k*_*e*(*L*)_/*k*_off_
β: *k*_out_/*k*_off_
γ: *k*_*e*(*RL*)_/*k*_off_

### Mathematical definitions

$A \mathop{=}\limits^{{\rm def}} B$: The symbol *A* is defined by the expression *B*
*f*(*x*) ∼ *g*(*x*) as *x* → ∞: *f*(*x*)/*g*(*x*)→1 as *x*→ ∞ (*x* → 0)
*f*(*x*) = *O*(ω(*x*)) as *x*→ ∞(0): There exist constants *K* > 0 and ξ > 0 such that
|*f* (*x*)| < *K*|ω(*x*)| if *x* > ξ (0 < *x* < ξ)

## Appendix 2: Short time analysis

We present a mathematical analysis for *Phase A* and show how ligand, receptor and ligand–receptor complex rapidly converge to the new *Plateau values* ${\overline L}, {\overline R}$ and ${\overline{RL}}$ given in (14), where we assume that *L*_0_ > *R*_0_.

In order to identify terms in the system (9) which dominate the dynamics in this initial phase, we define the dimensionless concentrations

62 $$ x=\frac{L}{L_0}, \quad y=\frac{R}{R_0}, \quad z=\frac{RL}{R_0} $$

and the dimensionless parameter μ = *R*_0_/*L*_0_. Substituting them into the system (1) we obtain

63 $$ \left\{ \begin{array}{l}  \frac{dx}{dt} = -k_{\rm on} R_0 x \cdot y + k_{\rm off} \mu z -  k_{e(L)}x \\  \frac{dy}{dt} = k_{\rm out}(1 - y) -k_{\rm on} L_0 x \cdot y+  k_{\rm off} z \\ \frac{dz}{dt} = k_{\rm on} L_0 x \cdot y - (k_{\rm off}+ k_{e(RL)})z\\ \end{array}\right. $$

Introducing the dimensionless time τ = *k*_on_ *R*_0_ *t*, then results in the dimensionless system

64 $$ \left\{ \begin{array}{l}  \frac{dx}{d \tau } = - x \cdot y + \varepsilon \mu z - \varepsilon \alpha x \\  \frac{dy}{d \tau } = \varepsilon \beta (1 - y) - \mu^{-1} x \cdot y+ \varepsilon z \\ \frac{dz}{d \tau } = \mu^{-1} x \cdot y - \varepsilon (1+\gamma) z \\ \end{array}\right. $$

where we have introduced the following dimensionless constants (see also (58)):

65 $$ \varepsilon = \frac{K_d}{R_0},\quad \alpha = \frac{k_{e(L)}}{k_{\rm off}}, \quad \beta = \frac{k_{\rm out}}{k_{\rm off}} \quad \hbox {and} \quad \gamma= \frac{k_{e(RL)}}{k_{\rm off}} $$

We multiply the third equation of (64) by μ and add the resulting equation to the first equation to obtain

66 $$  \frac{d}{d \tau } (x+\mu z) = - \varepsilon\left(\alpha x + \mu \gamma z\right) $$

Similarly, we add the second and the third equation to obtain

67 $$  \frac{d}{d \tau } (y+ z) = \varepsilon\left\{\beta (1-y) - \gamma  z\right\} $$

Since $\varepsilon \ll 1$ and $\varepsilon \alpha \ll1, \varepsilon \beta \ll1$ and $\varepsilon \gamma \ll1$ by Assumption B (cf. (13)), it follows that to good approximation,

68 $$ x(\tau )+\mu z(\tau ) = 1 \quad \hbox {and} \quad y(\tau )+z(\tau ) = 1 \quad \hbox {if} \, \varepsilon \tau \ll 1 $$

where we have used the fact that *x*(0) = 1, *y*(0) = 1 and *z*(0) = 0. Note that in light of the definition of τ and $\varepsilon,$ the assumption $\varepsilon \tau \ll 1$ is equivalent to *k*_off_ *t* ≪ 1.

### *Remark*

These approximate equalities state that the total amount of ligand (*L* + *RL*) and the total amount of receptor (*R* + *RL*) in the system remain more or less constant during this initial phase.

Using the expressions from (68) to eliminate *x* and *y* from the equation for *z* in (64), we obtain

69 $$ \frac{dz}{d \tau } = f(z) \stackrel{{\rm def}}{=} \frac{1}{\mu}(1-\mu z)(1-z) $$

where we have dropped the term $\varepsilon \mu (1+\gamma)z$ because $\varepsilon \mu \gamma < \varepsilon \gamma \ll1$ so that its impact is negligible.

Eq. (69) has two zeros: *z* = 1 and *z* = 1/μ > 1. Plainly *f*(*z*) > 0 for 0 ≤ *z* < 1 and for *z* > 1/μ, whereas *f*(*z*) < 0 for 1 < *z* < 1/μ. Since *z*(0) = 0 it follows that *z*(τ) increases and approaches the lower zero ${\overline z}=1$ as time tends to infinity:

70 $$ z(\tau ) \to {\overline z} =1\quad \hbox {as} \, \tau \to \infty $$

When we linearise Eq. (69) at *z* = 1, write $z(\tau ) = 1 + \zeta(\tau )$ and omit the terms involving $\zeta^n$ for *n* > 1 we obtain the equation

71 $$ \frac{d\zeta}{d\tau }\Big|_{z=1} = -\frac{1-\mu}{\mu}\zeta $$

Therefore,

72 $$ \tau _{1/2} = \ln(2) \times \frac{\mu}{1-\mu} \Longrightarrow t_{1/2} =\frac{\ln(2)}{k_{\rm on}(L_0-R_0)} $$

Thus, for $\varepsilon \tau \ll1,$ i.e., for *k*_off_ *t*_1/2_ to be small, as required in (68), the parameters and the initial ligand concentration need to be such that *k*_off_/(*k*_on_(*L*_0_ − *R*_0_) ≪ 1 or *L*_0_ − *R*_0_≫ *K*_*d*_.

In light of (68), the limit for *z*(τ) obtained in (70) implies the following limits for *x*(τ) and *y*(τ):

73 $$ x(\tau ) \to {\overline x} \mathop{=}\limits^{{\rm def}} 1-\mu{\overline z} = 1-\mu \quad \hbox {and} \quad y(\tau ) \to{\overline y} \mathop{=}\limits^{{\rm def}} 1- {\overline z} = 0\quad \hbox {as} \, \tau \to \infty $$

Returning to the original variables and writing ${\overline L} = L_0 {\overline x},$ ${\overline R} = R_0 {\overline y}$ and ${\overline{RL}} = R_0 {\overline z},$ we conclude that over a time span of *O*(1/(*k*_on_(*L*_0_ − *R*_0_)) we have

74 $$ L(t) \to {\overline L} = L_0-R_0, \quad R(t) \to {\overline R} = 0 \quad \hbox {and} \quad {\overline{RL}}(t) \to {\overline{RL}}= R_0 \quad\hbox {as}\, t \to \infty $$

## Appendix 3: Bounds of the receptor concentration

We establish two a-priori bounds: one for the total receptor concentration by *R*_tot_ = *R* + *RL* and one for the free receptor concentration *R*. Both bounds are *global* in time, i.e., they hold for all time.

### **Lemma 1**

*Suppose that* $R_{\rm tot}(0)= R_0 \mathop{=}\limits^{{\rm def}} k_{\rm in}/k_{\rm out}.$ *Then we have*:

75 $$ R_{\rm tot}(t) \le {\rm max}\left\{\frac{k_{\rm in}}{k_{\rm out}}, \frac{k_{\rm in}}{k_{e(RL)}}\right\} \quad \hbox{for}\, t \ge 0. $$

### *Proof*

Adding the last two equations of the system (1), we obtain

76 $$ \frac{dR_{\rm tot}}{dt} = k_{\rm in} - k_{\rm out} R - k_{e(RL)}RL $$

We now consider two cases: (I) *k*_*e*(*RL*)_ ≥ *k*_out_ and (II) *k*_*e*(*RL*)_ < *k*_out_

- **Case I** (*k*_*e*(*RL*)_ ≥ *k*_out_). We write Eq. (76) as
  77 $$ \frac{dR_{\rm tot}}{dt} = k_{\rm in} - k_{\rm out} R_{\rm tot} + (k_{\rm out} - k_{e(RL)}) RL $$
  and conclude that since, by assumption, *k*_*e*(*RL*)_ ≥ *k*_out_, we have
  78 $$ \frac{dR_{\rm tot}}{dt} \le k_{\rm in} - k_{\rm out} R_{\rm tot} $$
  Define the function $\rho_1(t) \mathop{=}\limits^{{\rm def}} R_{\rm tot}(t) - R_0.$ Then, transforming Eq. (78) and the initial value of *R*_tot_ to this new variable, we obtain
  79 $$ \frac{d\rho_1}{dt} \le - k_{\rm out} \rho_1, \quad \rho_1(0)=0 $$
  This implies that
  $$ \frac{d}{dt}\left(e^{k_{\rm out} t} \rho_1(t)\right) \le 0 \quad \hbox {and hence} \, \rho_1(t)\le \rho_1(0) = 0 $$
  Therefore,
  80 $$ R_{\rm tot}(t) \le R_0 = \frac{k_{\rm in}}{k_{\rm out}} $$
  as asserted. This completes the proof of the first inequality. $\square$
- **Case II** (*k*_*e*(*RL*)_ < *k*_out_). We now write Eq. (76) as
  81 $$ \begin{aligned} \frac{dR_{\rm tot}}{dt} &= k_{\rm in} - k_{e(RL)} R_{\rm tot} + (k_{e(RL)} - k_{\rm out}) RL \\ & \quad< k_{\rm in} - k_{e(RL)} R_{\rm tot} \\ \end{aligned} $$
  Put $\rho_2(t) \mathop{=}\limits^{{\rm def}} R_{\rm tot}(t) - R_*,$ where *R*_*_ = *k*_in_/*k*_*e*(*RL*)_, and note that by assumption, ρ_2_(0) = *R*_tot_(0) − *R*_*_ < 0. Proceeding as in the previous case we conclude that ρ_2_(*t*) < 0 for *t* ≥ 0 and hence that
  82 $$ R_{\rm tot}(t) < \frac{k_{\rm in}}{k_{e(RL)}} \quad \hbox {for all} \, t \ge 0 $$
  This completes the proof of the second inequality, and hence of the lemma. $\square$

### *Remarks*

1. The upper bound in Lemma 1 is independent of the amount of ligand that is supplied to the system and it is valid whether drug is given through a bolus administration or by means of a constant rate infusion.
2. Lemma 1 generalises an observation made before in [3, 15, 17], which states that
  83 $$ R_{\rm tot}(t) \equiv R_0 \quad \hbox {if and only if} \, k_{e(RL)} = k_{\rm out} $$
  Next, we prove bounds for *R*.

### **Lemma 2**

*Suppose that* *L*(0) = *L*_0_, *R*(0) = *R*_0_  *and* *RL*(0) = 0. *Then*

84 $$ \frac{R_0}{\displaystyle{1+\frac{k_{\rm on}}{k_{\rm in}}\frac{L_0}{R_0}}} < R(t) < R_0\left(1+K_d\frac{k_{\rm on}}{k_{\rm out}}\frac{L_0}{R_0}\right) \quad \hbox {for} \, 0 \le t < \infty $$

### *Proof*

From the conservation law (4) we deduce that *L*_tot_(*t*) is a decreasing function of *t*. Therefore,

85 $$ L(t) + RL(t) \le L_0 \quad \hbox {for all} \, t \ge 0 \Longrightarrow RL(t) \le L_0 $$

In order to prove the upper bound, we write

86 $$ \begin{aligned} \frac{dR}{dt} &= k_{\rm in} - k_{\rm out} R -k_{\rm on} L \cdot R + k_{\rm off} RL \\ &\quad< k_{\rm in} - k_{\rm out} R + k_{\rm off} RL \\ &\quad< (k_{\rm in} + k_{\rm off} L_0) - k_{\rm out} R = k_{\rm out} (R_+-R) \\ \end{aligned} $$

where

87 $$ R_+ = \frac{k_{\rm in}+k_{\rm off} L_0}{k_{\rm out}} $$

Plainly *R*(0) = *R*_0_ < *R*_+_, and we claim that *R*(*t*) < *R*_+_ for all *t* ≥ 0. Suppose to the contrary that there exists first a time *t*_0_ > 0 such that *R*(*t*_0_) = *R*_+_, i.e.,

88 $$ R(t)<R_+ \quad \hbox {for}\, 0<t<t_0 \quad \hbox {and} \quad R(t_0)=R_+ $$

We see that *R*(*t*) approached *R*_+_ from below as $t \nearrow t_0,$ so that *dR*/*dt* ≥ 0 at *t* = *t*_0_. However, we deduce from (86) that *dR*/*dt*(*t*_0_) < 0. Therefore, we have a contradiction and we may conclude that there exists no time *t*_0_ > 0 for which *R*(*t*) attains the value *R*_+_. This proves the claim and thereby the upper bound. $\square$

To prove the lower bound we write

89 $$ \begin{aligned} \frac{dR}{dt} &= k_{\rm in} - k_{\rm out} R-k_{\rm on} L \cdot R + k_{\rm off} RL \\ &\quad> k_{\rm in} - k_{\rm out} R -k_{\rm on} L \cdot R \\ &\quad> k_{\rm in} - (k_{\rm out}+ k_{\rm on} R_0) R = (k_{\rm out}+ k_{\rm on} R_0) (R_--R) \\ \end{aligned} $$

where

90 $$ R_- = \frac{k_{\rm in}}{k_{\rm out} + k_{\rm on} L_0} $$

Proceeding as with the upper bound, we establish the lower bound.

### **Corollary 1**

*Since* *R*_+_(*L*_0_) → *R*_0_ *and* *R*_−_(*L*_0_) → *R*_0_ *as* *L*_0_ → 0, *it follows that for any time* *t* ≥ 0,

91 $$ R (t) \to R_0 \quad \hbox {as} \, L_0 \to 0 $$

## Appendix 4: Derivation of the approximation *L*_approx_(*t*) and expressions for *AULC* and *CL*

We wish to solve the initial value problem

92 $$ \frac{dL }{dt} = - k_{e(L)}L - k_{\rm in}, \quad L(0)={\overline L} $$

where ${\overline L} \approx L_0 - R_0$ is the ligand concentration right after *Phase A*. We multiply this equation by $e^{k_{e(L)}t}$ to obtain

$$ \frac{d }{dt}(e^{k_{e(L)}t} L) = e^{k_{e(L)}t} k_{\rm in} $$

Integration over (0,*t*) yields the desired expression for the solution *L*_approx_(*t*) of Eq. (92):

93 $$ L_{\rm approx}(t) = ({\overline L} + \nu)e^{-k_{e(L)}t} - \nu, \quad \nu= \frac{k_{\rm in}}{k_{e(L)}} $$

Plainly, *L*(*t*) → − ν < 0 as $t \to \infty.$ Since *L*_approx_(*t*) is decreasing and *L*(0) > 0, it follows that there exists a unique time *T* ^*^ > 0 for which *L*_approx_(*t*) vanishes. An elementary computation yields the following expression for *T* ^*^:

94 $$ T^* = \frac{1}{k_{e(L)}}\ln\left(\frac{{\overline L}}{\nu}+1\right) = \frac{1}{k_{e(L)}}\ln\left(\frac{k_{e(L)}}{k_{\rm in}}{\overline L} +1\right) $$

For the three largest initial ligand concentrations used in case study this formula yields, as *L*_0_ decreases, respectively, *T* ^*^ ≈ 2818 h, *T* ^*^ ≈ 2042 h and *T* ^*^ ≈ 1372 h. This agrees well with the numerical results shown in Figs. 3, 4 and 5.

In order to derive the approximation for *AULC*(*L*_0_), we write

95 $$ \begin{aligned} \int\limits_0^{T^*}L_{\rm approx}(t) dt &= \frac{1}{k_{e(L)}} \int\limits_0^{k_{e(L)}T^*}\{({\overline L}+\nu)e^{-s}-\nu\} ds \\ &= \frac{1}{k_{e(L)}}\{({\overline L}+\nu)(1-e^{-k_{e(L)}T^*})- \nu k_{e(L)}T^*\} \end{aligned} $$

When we now substitute the expression for *T* ^*^ from (94) into (95), we obtain the desired formula:

96 $$ AULC(L_0) = \frac{1}{k_{e(L)}} \left\{L_0-R_0-\nu \log{\left(\frac{L_0-R_0+\nu}{\nu}\right)}\right\} $$

from which (28) follows. Applying the definition *CL*(*D*) = *D*/*AULC*(*D*) we obtain an expression for *CL*(*D*).

## Appendix 5: Small ligand asymptotics

When, as in *Phases C* and *D*, the ligand concentration drops to levels that are comparable to *K*_*d*_, a different scaling from the one used in Appendix 2 yields information about the relative importance of the different processes. For this range we define the scaling

97 $$ u=\frac{L}{K_d}, \quad v=\frac{R}{R_0}, \quad w=\frac{RL}{R_0} $$

which, after substitution into the system (9) yields

98 $$ \left\{ \begin{array}{l} \frac{du}{dt}= -k_{\rm on} R_0( u \cdot v - w) - k_{e(L)}u \\ \frac{dv}{dt}= k_{\rm out}(1-v) -k_{\rm off} (u \cdot v -w) \\ \frac{dw}{dt} = k_{\rm off} u \cdot v - (k_{\rm off}+ k_{e(RL)})w\\ \end{array}\right. $$

In addition we need to introduce a new, more appropriate, time scale for this phase. As we shall see, 1/*k*_off_ will be the right choice for this stage in the dynamics, and we define the new dimensionless time

99 $$ \tau = k_{\rm off} t $$

When we introduce this time variable into the system (98) we obtain

100 $$ \left\{ \begin{array}{l} \varepsilon \frac{du}{d\tau }= - u \cdot v + w - \varepsilon \alpha u \\ \frac{dv}{d\tau } = \beta(1-v) - (u \cdot v - w) \\ \frac{dw}{d\tau } = u \cdot v - (1+ \gamma)w \\ \end{array}\right. $$

where $\varepsilon, \alpha, \beta$ and γ are been defined as in (58) and (65):

101 $$ \varepsilon = \frac{K_d}{R_0},\quad \alpha = \frac{k_{e(L)}}{k_{\rm off}}, \quad \beta = \frac{k_{\rm out}}{k_{\rm off}} \quad \hbox {and} \quad \gamma = \frac{k_{e(RL)}}{k_{\rm off}} $$

Note that for the parameter values listed in Table 1, we have

$$ \varepsilon = 0.0009, \quad \alpha = 1.5, \quad \beta = 9, \quad \gamma = 3 $$

Thus, $\varepsilon$ is very small so that we may conclude from a standard singular perturbation argument [19, 20, 21] that after a very short period

102 $$ u \cdot v - w = 0 \quad \hbox {or} \quad L \cdot R = K_d\cdot RL $$

The equality (102) enables us to simplify the second and third equation of the system (100) to

103 $$ \frac{dv}{d\tau } = \beta(1-v) \quad \hbox {and} \quad \frac{dw}{d\tau } = - \gamma w $$

or, in terms of the original variables,

104 $$ \frac{dR}{dt} =k_{\rm out}(R_0-R) \quad \hbox {and} \quad\frac{dRL}{dt} = {- k_{e(RL)} RL} $$

Since at the start of *Phase C* we have *R* ≈ 0 and *RL* ≈ *R*_*_, we conclude that

105 $$ R(t) \approx R_0 (1- e^{-k_{\rm out}t}) \quad \hbox {and} \quad RL(t) \approx R_* e^{-k_{e(RL)}t} $$

## Appendix 6: Derivation of the steady state relations (47) and (48)

When we put the derivatives in the system (46) equal to zero and put $t_{\rm washout}=\infty,$ we obtain a nonlinear algebraic system of three equations involving the three variables *L*, *R*, *RL*. We eliminate *L* and *R* and derive the expression (47) for the steady state value of *RL*. Adding the first and the third equation of (46) yields a relation that involving only *RL* and *L*:

106 $$ k_f = k_{e(RL)}RL + k_{e(L)}L $$

Adding the second and the third equation of (46) yields a relation involving only terms of *R* and *RL*:

107 $$ k_{\rm in}= k_{e(RL)}RL + k_{\rm out}R $$

Using these two relations to eliminate *R* and *L* from the first equation, we obtain a quadratic equation for *RL*:

108 $$ X^2 + (k_f + k_{\rm in} + q)X + k_f k_{\rm in}= 0, \quad X=k_{e(RL)}RL $$

where *q* = (*k*_*e*(*L*)_/*k*_*e*(*RL*)_)*k*_out_ *K*_*m*_. Plainly, it has two distinct roots, *RL*_+_ and *RL*_−_ and they are endowed with the property

109 $$ k_{e(RL)}(RL_+ + RL_-) = k_f + k_{\rm in} + q $$

Adding (106) and (107) we deduce that the steady state value of *RL* must satisfy the inequality

110 $$ 2 k_{e(RL)}RL < k_f + k_{\rm in} $$

This implies that the desired steady state value of *RL* must be given by the smaller of the two roots: *RL*_−_. The two expressions (106) and (107) then yield the corresponding steady state values for *L* and *R*.

## Appendix 7: Computation of the terminal slope

In order to compute the terminal slope λ_*z*_ for the ligand concentration profile we linearise the system (9) about the steady state (*L*, *R*, *RL*) = (0, *R*_0_, 0). Writing *L* = ξ, *R* = *R*_0_ + η and $RL=\zeta,$ we obtain the linear system

111 $$ \left\{ \begin{array}{l} \frac{d\xi}{dt} = -(k_{\rm on} R_0 + k_{e(L)}) \xi + k_{\rm off} \zeta \\ \frac{d\eta}{dt} = -k_{\rm on} R_0 \xi - k_{\rm out} \eta + k_{\rm off} \zeta \\ \frac{d\zeta}{dt} = k_{\rm on} R_0 \xi - (k_{\rm off}+ k_{e(RL)})\zeta\\ \end{array}\right. $$

when higher order terms are omitted. It is convenient to write this system in vector- and matrix notation:

112 $$ \frac{dY}{dt}=AY \quad \hbox {where} \quad Y=\left(\begin{array}{l} \xi \\ \eta \\ \zeta \\ \end{array}\right) $$

and *A* is the coefficient matrix of the linear system (111):

113 $$ A= \left(\begin{array}{lll} -(k_{\rm on}R_0+ k_{e(L)}) & 0 & k_{\rm off} \\ -k_{\rm on}R_0 & -k_{\rm out} & k_{\rm off} \\ k_{\rm on}R_0 & 0& - (k_{\rm off} +k_{e(RL)}) \\ \end{array}\right) $$

We assume that the matrix −*A* has three distinct eigenvalues λ_1_, λ_2_ and λ_3_ and that *Y*_1_, *Y*_2_ and *Y*_3_ are the corresponding eigenvectors. The *General Solution* of Eq. (112) then takes the form

114 $$ Y(t) = C_1 Y_1 e^{-\lambda_1t} + C_2 Y_2 e^{-\lambda_2t} + C_3 Y_3 e^{-\lambda_3t} $$

where *C*_1_, *C*_2_ and *C*_3_ are arbitrary constants.

The eigenvalues λ_*i*_ (*i* = 1, 2, 3) are the roots of the equation

$$ {\rm det}(A+\lambda I)=0 $$

Thus, we find that

$$ \lambda_1 = k_{\rm out} $$

and λ_2_ and λ_3_ are the roots of the quadratic equation

115 $$ \lambda^2 - a \lambda + b=0 $$

where

116 $$ \begin{aligned} a& = k_{\rm on}R_0 + k_{e(L)} + k_{\rm off} +k_{e(RL)} = k_{\rm on}R_0\left\{1+ \varepsilon \alpha+ \varepsilon (1+\gamma)\right\} \\ b& = k_{\rm on}R_0 k_{e(RL)}+ k_{e(L)} k_{\rm off} + k_{e(L)} k_{e(RL)} = k_{\rm on}R_0 k_{e(RL)}\left(1+ \varepsilon \frac{\alpha}{\gamma}(1+\gamma) \right) \end{aligned} $$

and $\varepsilon, \alpha, \beta$ and γ are defined in Eqs. (58) and (64). Since $\varepsilon \ll 1$ and α, β and γ are of moderate size (cf. Assumptions A and B in (13)), we have to good approximation,

117 $$ a = k_{\rm on}R_0 \quad \hbox {and} \quad b= k_{\rm on}R_0 k_{e(RL)} $$

This implies that $b/a^2 = \varepsilon \gamma \ll 1,$ so that we conclude that

118 $$ \lambda_1 = k_{\rm out}=0.0089, \quad \lambda_2 = a=k_{\rm on} R_0 = 0.11 \quad \hbox {and} \quad \lambda_3 = \frac{b}{a} = k_{e(RL)} = 0.003 $$

i.e., the terminal slope λ_*z*_ is given by λ_*z*_ = *k*_*e*(*RL*)_ = 0.003. This agrees with the numerical value found in Figs. 5, 6, 7 and 8. For an earlier derivation we refer to [25].

### *Remark*

Naturally, when *k*_*e*(*RL*)_ = 0 then ligand will still leak out of the system, albeit more slowly, thanks to direct elimination of ligand. For this case we obtain the following terminal slope:

119 $$ \lambda_z = \frac{b}{a} = k_{e(L)} \frac{K_d}{R_0}. $$

## Footnotes

1 For the definition of the *O*-symbol, see Appendix 1.
